# Gating mechanism of Kv11.1 (hERG) K^+^ channels without covalent connection between voltage sensor and pore domains

**DOI:** 10.1007/s00424-017-2093-9

**Published:** 2017-12-21

**Authors:** Pilar de la Peña, Pedro Domínguez, Francisco Barros

**Affiliations:** Departamento de Bioquímica y Biología Molecular, Edificio Santiago Gascón, Campus de El Cristo, Universidad de Oviedo, 33006 Oviedo, Asturias Spain

**Keywords:** Potassium channel, Split channel, Gating, hERG, Structure-function relationship

## Abstract

Kv11.1 (hERG, *KCNH2*) is a voltage-gated potassium channel crucial in setting the cardiac rhythm and the electrical behaviour of several non-cardiac cell types. Voltage-dependent gating of Kv11.1 can be reconstructed from non-covalently linked voltage sensing and pore modules (split channels), challenging classical views of voltage-dependent channel activation based on a S4–S5 linker acting as a rigid mechanical lever to open the gate. Progressive displacement of the split position from the end to the beginning of the S4–S5 linker induces an increasing negative shift in activation voltage dependence, a reduced *z*_*g*_ value and a more negative Δ*G*_0_ for current activation, an almost complete abolition of the activation time course sigmoid shape and a slowing of the voltage-dependent deactivation. Channels disconnected at the S4–S5 linker near the S4 helix show a destabilization of the closed state(s). Furthermore, the isochronal ion current mode shift magnitude is clearly reduced in the different splits. Interestingly, the progressive modifications of voltage dependence activation gating by changing the split position are accompanied by a shift in the voltage-dependent availability to a methanethiosulfonate reagent of a Cys introduced at the upper S4 helix. Our data demonstrate for the first time that alterations in the covalent connection between the voltage sensor and the pore domains impact on the structural reorganizations of the voltage sensor domain. Also, they support the hypothesis that the S4–S5 linker integrates signals coming from other cytoplasmic domains that constitute either an important component or a crucial regulator of the gating machinery in Kv11.1 and other KCNH channels.

**Electronic supplementary material:**

The online version of this article (10.1007/s00424-017-2093-9) contains supplementary material, which is available to authorized users.

## Introduction

Voltage-gated potassium (Kv) channels play a crucial role for regulation of excitability in numerous cell types [3, 24, 63]. Kv channels [21] belong to the voltage-gated ion channel superfamily that also includes the voltage-gated sodium (Na_v_) and calcium (Ca_v_) channels, the cyclic nucleotide-gated (CNG) and the hyperpolarization-activated cyclic nucleotide-gated (HCN) channels and the transient receptor potential (TRP) channels [24, 27, 65]. Of these, the Kv11.1 (hERG, KCNH2) channel mediates the cardiac potassium current IKr, involved in the repolarization of the cardiac action potential and playing a crucial role to prevent arrhythmias induced by early after depolarizations or ectopic beats, such that mutations in Kv11.1 lead to inherited type 2 long QT syndrome. This channel is also the molecular target of most drugs that cause arhythmias. Kv11.1 also plays a key role in setting the electrical behaviour of a variety of non-cardiac cell types (reviewed in [2, 38, 42, 54]). These physiological roles of Kv11.1 derive from an unusual combination of gating kinetics including a slow rate of activation, a very fast voltage-dependent inactivation and inactivation recovery and a particularly slow deactivation on repolarization [2, 37, 42, 54].

It is known that Kv channels are tetrameric, with each subunit being formed by two structural and functional modules: a pore domain (PD) or permeation module constituted of the transmembrane segments S5–S6 and an intervening pore loop and a voltage sensing domain (VSD) consisting of transmembrane segments S1 to S4, both modules being linked through the so-called S4–S5 linker [4, 46, 63]. Classical views of voltage-dependent Kv gating invoked a mechanism based in the S4–S5 linker acting as a rigid mechanical lever to open the gate located at the bottom of transmembrane helix S6, following the voltage sensor reorganizations triggered by changes in transmembrane voltage [5, 55]. However, we have recently shown that for KCNH family channels such as Kv10.1 (KCNH1, Eag1) and Kv11.1, voltage-dependent gating can be reconstructed from non-covalently linked VSD and PD modules (split channels [33]). Further supported by more recent structural evidence [60, 62], these data point to an alternate gating mechanism for these channels diverging from that operating in the more traditionally considered *Shaker*-like Kv channels, in which the coupling of the VSD and the PD is achieved in an “electrointeractional” way involving some specific regions of both modules [11–13, 33, 62]. However, the exact nature of these interactions and, particularly, their dynamics during the gating process(es) remain to be established, and the use of split channels could constitute an excellent option for their study. Indeed, it has been repeatedly proposed that in Kv11.1 and other members of the KCNH family, the S4–S5 linker can act as an integrator of signals coming from other cytoplasmic channel domains [1, 35, 47, 67] and a direct contact between the Kv11.1 N-tail and the S4–S5 and the C-linkers has been demonstrated [11–13, 16] and recently visualized in the cryo-EM structure of Kv11.1 [60]. Thus, in this work, we studied how different structural alterations in the split interface between the VSD and the PD modules influence the functional behaviour of Kv11.1 split channels. Our results demonstrate that moving the split point along the sequence of the S4–S5 linker gradually modifies the gating characteristics of the split channels. Interrupting the covalent connection at the N-terminal end of the S4–S5 linker leads to a destabilization of the split closed state(s), providing functional evidence that supports the proposal that this region non-covalently interacts with the PD gate to open and close the channel [33, 62]. Our data also suggest that by actively pushing the C-terminal end of the S4 helix and/or the initial section of the S4–S5 linker against the S5–S6 module, the closing of an otherwise intrinsically stable open pore can be favoured at negative transmembrane potentials. This process is also influenced by other cytoplasmic structures such as the highly flexible N-tail of the VSD, that allosterically could affect different phases of a non-mechanical, but allosteric mechanism of gating [33].

## Materials and methods

### Molecular biology, mutagenesis and expression in *Xenopus laevis* oocytes

Kv11.1 split channels were generated as PCR fragments containing the desired coding sequences that were inserted into the pSP64A+ vector as HindIII-BamHI fragments. The N-terminal demi-channel fragment for split 539 was synthesized using a sense oligonucleotide containing a HindIII site, a Kozak’s signal and the sequences for the initial eight Kv11.1 residues together with the corresponding antisense oligonucleotide carrying the coding sequence for residues 529 to 539, followed by a stop codon and the BamHI recognition site. For the C-terminal demi-channel fragment synthesis, the sense oligonucleotide was designed to contain a HindIII site, a Kozak’s sequence and the start codon followed by the 540 to 550 Kv11.1 coding sequence, whereas the antisense oligonucleotide covered the last 10 residues of the protein (1049–1059), a stop codon and the BamHI recognition sequence. Subsequent split channels were generated in an identical manner, but to modify the N-terminal of the constructs, the antisense oligonucleotide was designed to contain the desired Kv 11.1 final coding sequence before the stop codon. In the case of the C-terminal constructs, the sense oligonucleotide was designed to contain the corresponding coding sequences of the different C-terminal demi-channels after the start codon.

Split channel variants bearing different N-terminal deletions were also created by PCR with the desired deletions introduced in the sense oligonucleotide. Split channel constructs with the indicated single point mutations were created by overlapping PCR as previously described [11–13]. All constructs were analysed by standard fluorescence-based DNA sequencing to confirm the mutations and verify the absence of errors.

Procedures for frog anesthesia and surgery to obtain oocytes and microinjection have been detailed elsewhere [1, 3, 11–13, 56]. Oocytes were maintained in OR-2 medium (82.5 mM NaCl, 2 mM KCl, 2 mM CaCl_2_, 2 mM MgCl_2_, 1 mM Na_2_HPO_4_, 10 mM HEPES, at pH 7.5). Cytoplasmic microinjections were performed with 50 nl of in vitro synthesized cRNA per oocyte.

### Electrophysiological recording and analysis

Two-electrode voltage clamp recordings were performed as previously described [1, 3, 11–13, 56] in manually defolliculated oocytes at room temperature 2–3 days after injection, using a Turbo TEC-01C amplifier (NPI electronics). The intracellular electrodes had resistances of 0.4–0.8 MΩ when filled with 3 M KCl. Unless otherwise stated, recordings were obtained in OR-2 medium. In some cases, high-K^+^ OR-2 medium in which 50 mM KCl replaced an equivalent amount of NaCl was used to maximize currents of those constructs showing a low level of functional expression. Oocytes showing membrane potentials more positive than − 30 mV after impalement with the first electrode in OR-2 medium were discarded. Current recordings were obtained in an experimental chamber of 0.12 ml volume continuously perfused at 2.4 ml/min. For experiments with [2-(trimethylammonium)ethyl]methanethiosulfonate chloride (MTSET; Biotium), the reagent was aliquoted in powder, stored at − 20 °C and dissolved in OR-2 just before application to each individual cell. In this case, silver chloride ground electrodes were connected to the bath chamber through agar bridges. Data acquisition and analysis were performed with the Pulse-PulseFit (HEKA Electronics) and IgorPro (WaveMetrics) software packages running on Macintosh computers. Ionic currents sampled at 1 or 10 kHz were obtained using the voltage protocols indicated in the graphs.

The voltage dependence of activation was assessed by standard tail current analysis using depolarization pulses of variable amplitude. For very rapidly deactivating constructs, fitting the relaxation of the tail currents and extrapolating the magnitude of the decaying current to the time the depolarizing pulse ended were used to determine the amount of current passing through channels opened on depolarization without the influence of rapid inactivation. Tail current magnitudes normalized to maximum were fitted with a Boltzmann function to estimate the *V*_1/2_ and equivalent gating (*z*_*g*_):

$$ {I}_{\mathrm{tail}}/{I}_{\mathrm{max}}=1/\left[\right(1+\exp \left(\left({V}_{1/2}-V\right){z}_gF/ RT\right)\Big] $$

where *V* is the test potential and *F*, *R* and *T* are Faraday constant, gas constant and absolute temperature, respectively. This function was also used to fit the MTSET voltage dependence data. The activation data were also fitted with a second Boltzmann function:

$$ {I}_{\mathrm{tail}}/{I}_{\mathrm{max}}=1/\left[\right(1+\exp \left(\left(\Delta {G}_0-{Vz}_gF\right)/ RT\right)\Big] $$

where Δ*G*_0_ is the work done at 0 mV. Although both equations are equivalent, from the last expression, the effect of every mutation on changes in the chemical potential (Δ*G*_0_) and electrostatic potential (*− Vz*_*g*_ *F*) that drives activation can be obtained.

The time course of voltage-dependent activation was studied using an indirect envelope-of-tail currents protocol, varying the duration of depolarization prepulses, and following the magnitude of the tail currents on repolarization. The time necessary to reach a half-maximal tail current magnitude was used to compare the speed of activation of the different channels.

The rates of deactivation were determined from negative amplitude biexponential fits to the decaying phase of tail currents using a function

$$ y={A}_f\exp \left(-\mathrm{inv}{\tau}_f\times x\right)+{A}_{\mathrm{s}}\exp \left(-\mathrm{inv}{\tau}_s\times x\right)+C $$

where *τ*_*f*_ and *τ*_*s*_ are the time constants of fast and slow components, *A*_*f*_ and *A*_*s*_ are the relative amplitudes of these components and *C* is a constant. In this case, the first cursor of the fitting window was advanced to the end of the initial hook because of the recovery of inactivation.

Onset of fast inactivation was studied after activation and inactivation of the currents with a prepulse to positive voltages, followed by a second short prepulse to around − 100 mV used to recover the channels from inactivation, and a subsequent test pulse to different voltages to reinactivate the channels. Time constants for the onset of inactivation were obtained from current traces fitting a single-exponential function to the decaying portion of the currents during the test pulses. The voltage dependence of inactivation was determined with an alternative triple pulse protocol in which cells were depolarized to + 40 mV for several seconds to activate/inactivate the channels and subsequently allowed to relax to a inactivation steady state during a brief test pulse at different voltages, followed by a third step to + 40 mV in which the initial current magnitude was measured to assess the relative number of channels available to activate at the end of the test pulse. Due to the fast deactivation at negative voltages during the test pulses, the closing rates were obtained in every oocyte from biexponential fits to the decaying tail currents as indicated above. These rates were used to determine the proportion of channels closed at the end of every test pulse and to correct for closing-induced decreases in the initial current magnitude at the beginning of the third step [43, 54].

For experiments designed to study the accessibility of engineered S4 cysteines to MTSET, we routinely used a brief voltage ramp as a stimulatory step that allowed us to kinetically follow the possible shifts in MTSET-induced voltage dependence, either repetitively pulsing the cells at 5-s intervals or maintaining them without pulsing at the indicated holding potentials. All constructs used for this purpose contained a Cys introduced in position 521 of the upper S4 helix (to follow its modification in the presence of MTSET) and two additional mutations (C445V and C449V) to prevent any inadvertent effect caused by MTSET modification of the endogenous cysteines of the Kv11.1 S1–S2 linker. Due to the differences exhibited by different splits in the magnitude of the shift in voltage dependence or in the extent of closing impairment induced by the MTS reagent, the rates and/or the voltage dependence of modification were subsequently obtained quantifying (i) the magnitude of the peak current increase during the ramp; (ii) the decrease in the time necessary to reach the current peak during the ramp; (iii) the change in the amount of current recorded at the end of a conditioning voltage step to negative voltages (e.g. − 120 mV), included immediately before or after the ramp; and (iv) the decrease in the ratio of the slopes (rectification factor) obtained from the current traces during the steeper raising phase and the minimum slope phase, both in the middle and at the beginning of the ramps, respectively. It is important to emphasize that the relatively slow time course of MTSET effects in both the continuous and different split channels with the I521C mutation is not due to any constraints imposed by our measuring system. Thus, (i) collapse of the cell membrane potential by a high K^+^ extracellular OR-2 solution was completed in a few seconds, and (ii) the time constants of MTSET effects during the test ramps were around 30% smaller in continuous channels carrying a more extracellularly exposed cysteine at position 520 instead of 521 [14].

### Statistics

Data values given in the text and in figures with error bars represent the mean ± SEM for the number of indicated cells. Comparisons between data groups were at first performed by parametric Student’s impaired *t* test (two-tailed). When significant differences in standard deviation were present, an alternate Welch’s test or non-parametric Wilcoxon or Mann-Whitney test were also used. In all cases, *p* values < 0.05 were considered as indicative of statistical significance.

## Results

### Gradual alterations in activation voltage dependence of Kv11.1 channels split at different positions along the S4–S5 linker

**Fig. 1:**
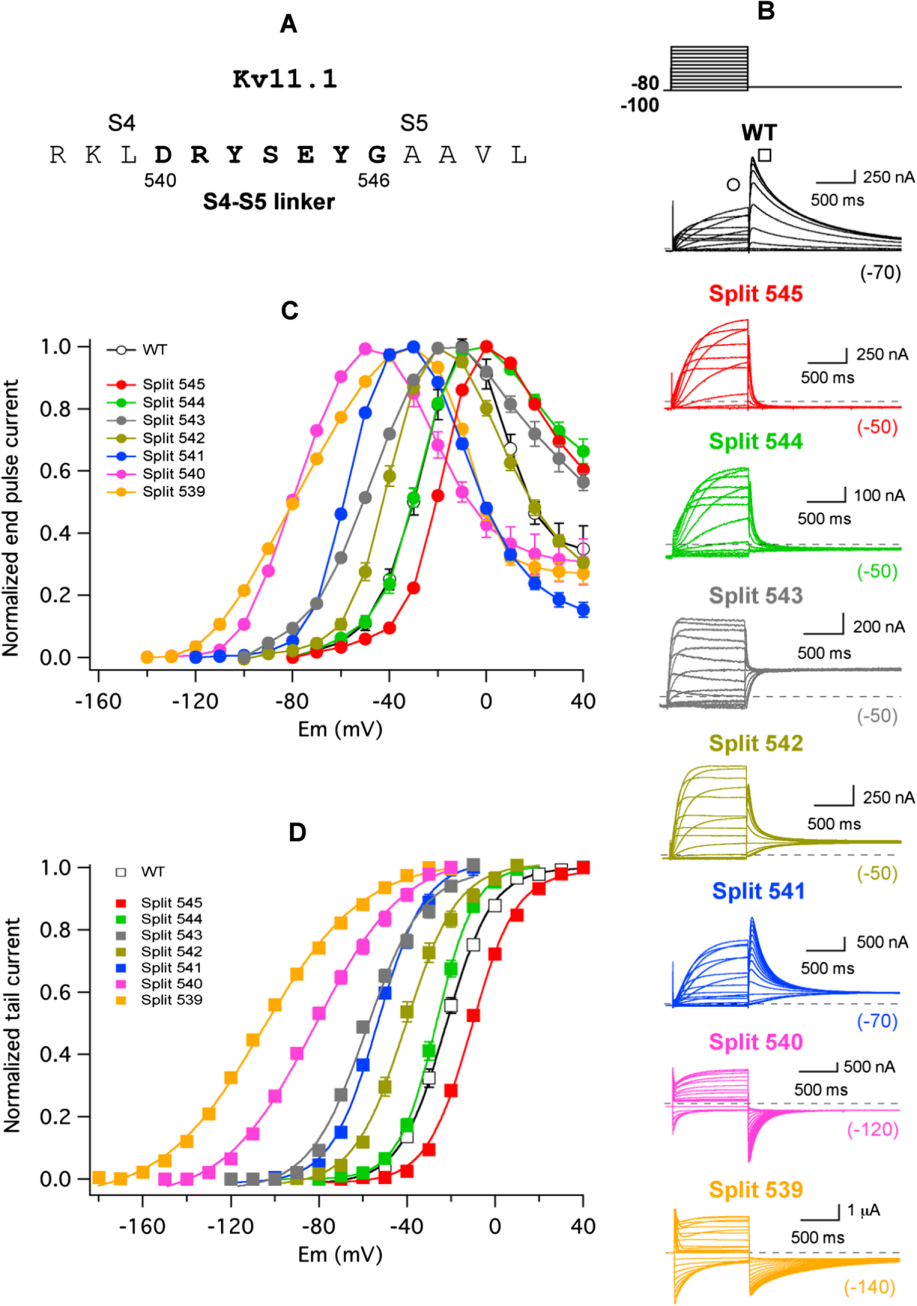
Effect of split position displacement on activation voltage dependence of Kv11.1 split channels. **a** Kv11.1 sequence of the S4–S5 linker region. Linker boundaries are chosen based in the structural information provided by the cryo-EM architecture of Kv11.1 [60]. **b** Representative membrane currents from individual oocytes expressing the indicated channel variants submitted to 1-s depolarization pulses at different potentials at 10 mV intervals form a holding potential of − 80/− 100 mV, followed by a repolarization step as indicated at the top. Note the different repolarization potentials for different constructs as enclosed in parentheses at the end of the current traces. Currents recorded without leak subtraction are shown. WT continuous wild type. **c** Averaged *I* versus *V* relationships measured at the end of the depolarization step (signalled by the circle in the WT traces of **b**). Note the typical Kv11.1 *n*-shaped curves due to the strong rectification as a result of the slow activation and fast inactivation overlap at positive voltages. **d** Plots of normalized peak tail current magnitudes (square in the WT current traces of **b**) as a function of depolarizing voltage. Continuous lines are Boltzmann fits to the data as indicated in “Materials and methods” section

Expression of Kv11.1 VSD and PD as separate polypeptides (demi-channels) in *Xenopus* oocytes yields functional voltage-gated ion channels (split channels) strikingly similar to continuous Kv11.1 [33]. More recent structural evidence with Kv11.1 [60] and the highly homologous Kv10.1 [62] indicates that the S4–S5 linker is much shorter in these channels, extending from the base of the S4 transmembrane helix at residue D540 in Kv11.1 to the beginning of S5 helix at around G546 (Fig. 1a). Since the Kv11.1 Y545 split channel showed most of the voltage-dependent properties of the continuous protein [33], these results challenged the traditional view of the S4–S5 loop acting as a mechanical lever to transduce the voltage-dependent motions of the VSD to the cytoplasmic gate at the bottom of S6. To obtain some additional insights about the mechanism involved in the coupling of the voltage sensor to the pore module in the presence of an interrupted S4–S5 linker, we generated a family of split channels in which the covalent link between the VSD and the PD was sectioned at different residues along the S4–S5 loop. As shown in Fig. 1b–d, apart from the previously described Y545 split, all channels split after residues L539, D540, R541, Y542, S543 and E544 gave rise to voltage-dependent currents following heterologous expression in *Xenopus* oocytes studied under two-electrode voltage clamp conditions. As reported for the case of the isolated demiN-1/545 and demiC-546/1159 components of the Y545 split [33], no detectable currents were observed when only one of the channel halves of any split was separately expressed in the oocytes. We first compared in the different split channels the voltage dependence of current activation after 1-s test pulses to different voltages followed by a hyperpolarization to negative potentials (Fig. 1b). Strikingly, as shown in the plots of normalized currents measured both at the end of the depolarizing step (Fig. 1c) and at the peak of the tail elicited during the hyperpolarizing pulse (G/V plots; Fig. 1d), the potential for half-maximal isochronal activation of the different constructs was gradually shifted to the left when the split point was displaced from position 545 to 539. Additionally, a significantly smaller amount of equivalent gating charges (*z*_*g*_ values) estimated from the slope of the G/V curves was observed in those constructs interrupted at the S4 helix/S4–S5 linker interface. Thus, Boltzmann fits (see “Materials and methods” section) to the normalized tail current data yielded *V*_1/2_ and *z*_*g*_ values of − 22.2 ± 0.9 mV/2.5 ± 0.03 (WT, *n* = 6), − 10.6 ± 0.8 mV/2.5 ± 0.08 (split 545, *n* = 8), − 24.6 ± 0.9 mV/2.0 ± 0.03 (split 544, *n* = 13), − 57.5 ± 0.7/2.4 ± 0.1 (split 543, *n* = 11), − 42.3 ± 0.9 mV/2.2 ± 0.07 (split 542, *n* = 13), − 55.4 ± 0.3 mV/2.3 ± 0.06 (split 541, *n* = 6), − 79.3 ± 1.9 mV/1.7 ± 0.05 (split 540, *n* = 8) and − 100.7 ± 1.1 mV/1.3 ± 0.03 (split 539, *n* = 6). Interestingly, Fig. 1c also shows that the inward rectification properties of the channels at positive voltages remained similar in all cases, suggesting a basically unaltered voltage-dependent inactivation behaviour of the different splits. Indeed, as illustrated in Suppl. Fig. 1 for the two most different splits of the series (splits 545 and 539), no significant changes with respect to the wild-type continuous channels were observed in the voltage dependence of inactivation when directly measured with a triple pulse voltage protocol ([43, 54], see “Materials and methods” section). Time constants for the development of inactivation were unaltered for the same constructs (see also [33]), when studied with a second triple pulse protocol as also detailed elsewhere ([43, 54, 56], see “Materials and methods” section). Although the S4 helix of Kv11.1 acts as the voltage sensor for both activation and inactivation gating, different regions of the sensor and a quite distinct molecular mechanism are involved in both gating processes [38, 40]. This could explain why excisions within the S4–S5 linker are able to produce alterations in activation/deactivation gating and voltage-dependent structural VSD reorganizations (see also below), without an impact on the voltage-dependent Kv11.1 inactivation. Finally, fitting the activation voltage dependence of the peak tail current data with a Boltzmann function that separates the chemical (Δ*G*_0_) and electrostatic (− *z*_*g*_ *EF*) components of the free energy changes necessary to open the channels (see “Materials and methods” section) indicated that the Δ*G*_0_ values of the splits became increasingly negative as the excision point moved from Y545 to L539. Thus, the Δ*G*_0_ values were − 1270 ± 45 cal/mol for the WT channel and − 580 ± 45, − 780 ± 36, − 2722 ± 207, − 1900 ± 122, − 2500 ± 91, − 3075 ± 129 and − 3073 ± 58 cal/mol for splits Y545, E544, S543, Y542, R541, D540 and L539, respectively. This indicates that in the absence of a transmembrane electric field, the equilibrium is gradually shifted towards the open state when the split point is displaced from position 545 to 539. Therefore, changes observed both in the *V*_1/2_ shifts and the ΔG_0_ values would be consistent with an increased destabilization of the closed state, a stabilization of the open state or both, following displacement of the breaking point towards the base of transmembrane helix S4.

### Destabilization of the closed state in Kv11.1 channels split at the S4 helix/S4–S5 linker boundary

**Fig. 2:**
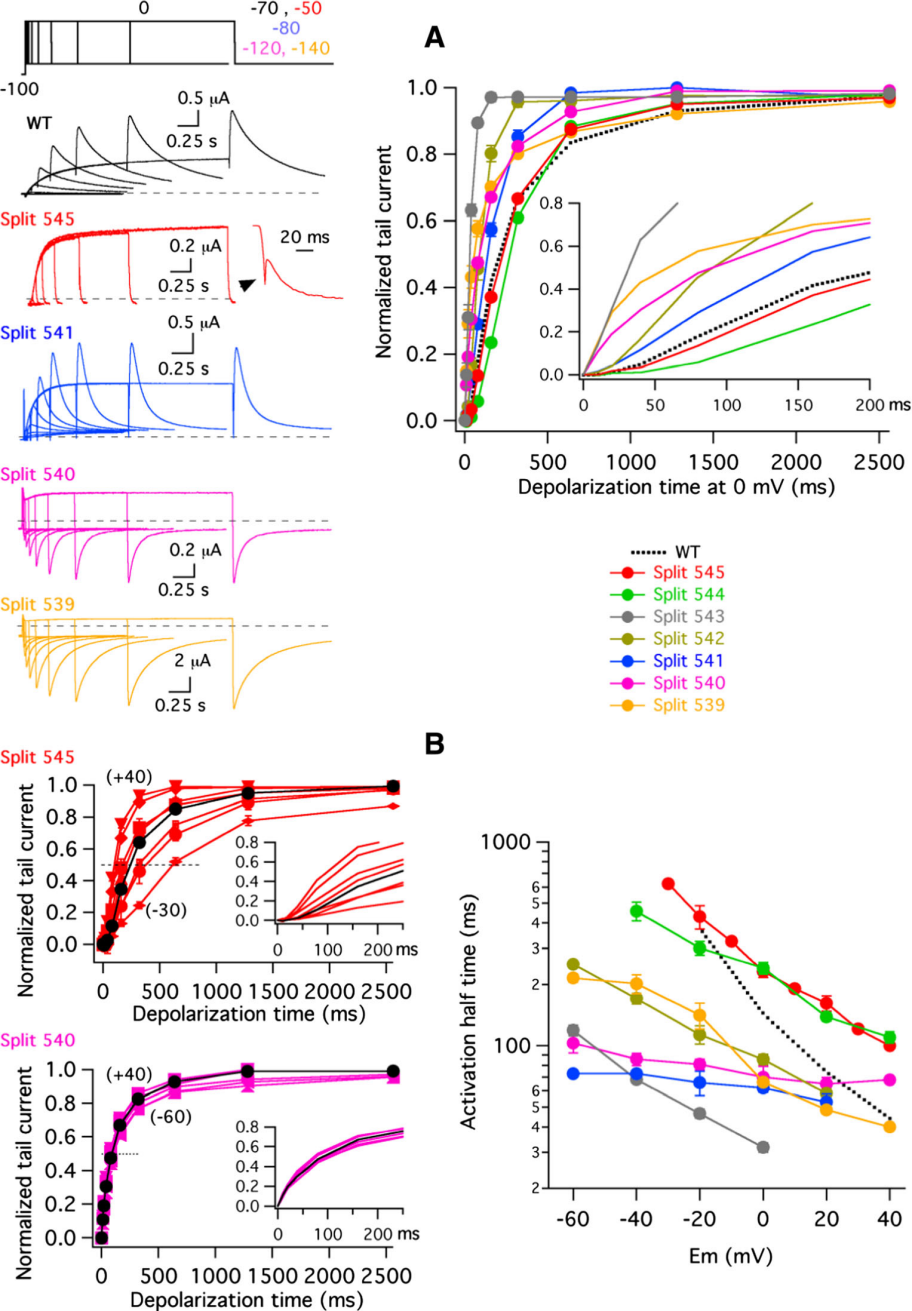
Effect of split position displacement on Kv11.1 split voltage-dependent activation rates. **a** Comparison of time course of current activation at 0 mV. Families of representative membrane currents from individual oocytes (left). The duration of a depolarizing prepulse to 0 mV was varied as illustrated in the envelope-of-tail currents protocol at the top, illustrative of that used with WT channels, followed by a repolarization step to the indicated potentials (− 70, − 50, − 80, − 120 and − 140 mV for WT, split 545, split 541, split 540 and split 539 variants, respectively). Cells expressing split 539 and split 540 channels were held at − 140 and − 120 mV trying to ensure that they also start in deactivated state. An enhanced view of the very rapidly deactivating split 545 tail current at the end of the 2560-ms depolarizing step is shown in the inset. Averaged plots of normalized tail current magnitudes versus depolarization time at 0 mV (right). Values from continuous wild-type channels are shown as a dotted line for comparison. An expansion of the initial 200 ms to highlight the progressive disappearance of the early current delay in the sigmoidal activation time course is shown in the inset. **b** Comparison of activation rate voltage dependence. Plots of normalized tail current magnitudes versus depolarization times at voltages between − 30 and + 40 mV (split 545) or − 60 and + 40 mV (split 540) at 10 and 20 mV intervals were generated from current traces as indicated in **a** (left). These plots were used to measure times necessary to attain half-maximum current magnitudes (dotted lines). Expanded views of the initial 250 ms of the plots are shown in the insets. Data obtained at 0 mV are represented in black. Dependence of activation rates on depolarization membrane potentials (right). The time necessary to attain half-maximum tail current magnitude is plotted versus depolarization potential. Values from continuous wild-type channels are shown as a dotted line for comparison

We next checked the impact of changing the split position on channel activation rates. It is known that activation of Kv11.1 constitutes a multi-step sequential process in which the final and voltage-dependent closed-to-open transition is preceded by several closed states [6, 18, 30, 41, 45, 54, 57]. This leads to a sigmoidal activation time course characterized by an initial delay preceding the exponential latter half of the current records. We used an envelope-of-tails voltage protocol in order to analyse the activation rates and to evaluate the proportion of channels activated during a depolarization step without the contribution of the inactivation process [3, 32, 51]. Data obtained with different constructs analysed by this method are compared in Fig. 2. Using the depolarization at 0 mV as a reference, it is observed that the activation time course of a Y545 split channel remains analogous to that of the uncut wild-type channel (Fig. 2a). However, the characteristic initial delay is progressively reduced by moving the split point from position 545 to 539; indeed, no delay is observed in the activation time course of the splits D540 and L539 (see inset of Fig. 2a). Figure 2b shows data obtained over a more extensive voltage range from the analysis of the different splits. Interestingly, unlike the results obtained with split Y545, a complete absence of initial delay is observed in split D540 even at a depolarization level of − 60 mV (left panels of Fig. 2b). The time course could still be accurately studied at this level due to the negatively shifted activation voltage dependence exhibited by this split channel. Comparisons of activation rates for all the splits using the time necessary to attain half-maximum tail current magnitude at every depolarization potential are illustrated in the right panel of Fig. 2b. Slightly slower activation kinetics than those of wild-type channels were induced by interruptions at the end of the S4–S5 linker (splits Y545 and E544). However, faster rates were observed with the rest of the splits. Also, a particularly reduced effect of the depolarization potential on the activation rates was observed in some of the splits near the S4 transmembrane segment (e.g. splits D540 and R541). Altogether, these results suggest that those channels disconnected near the carboxy terminus of the S4 helix present a reduced ability to reach more distal closed state(s) and therefore support the conclusion that an increased destabilization of the closed state(s) is induced when the excision point of the splits is displaced towards position 539. The inability to move through the voltage-dependent transitions towards the initial closing state(s) [18, 41, 54, 57] would be also consistent with the decreased *z*_*g*_ values exhibited by the near-S4 splits. Furthermore, the accelerated activation tendency caused by moving the split point from position 545 to 539 also suggests that some specific chemical interactions that raise the energy barrier for activation are progressively disrupted when the split point moves towards the base of the S4 segment.

### Effect of S4–S5 linker interruptions on deactivation kinetics of Kv11.1 channels

**Fig. 3:**
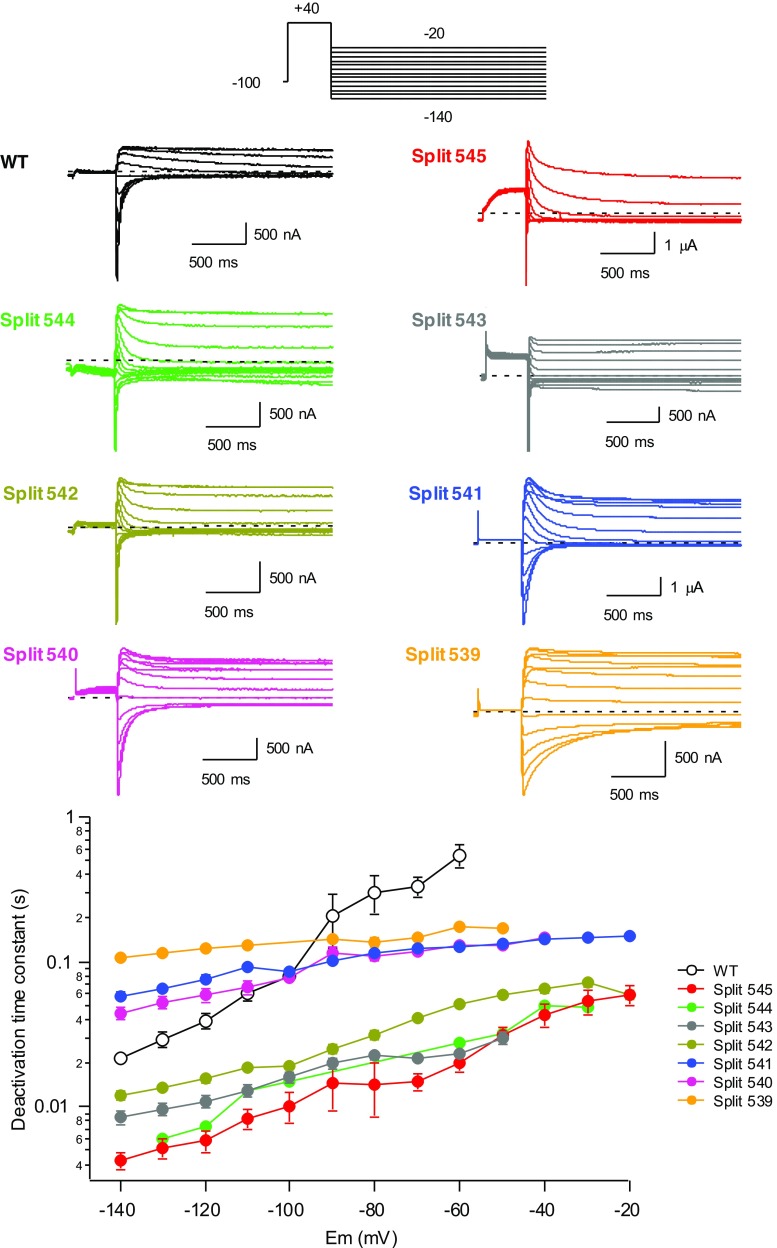
Effect of split position displacement on voltage-dependent deactivation kinetics of Kv11.1 splits. Representative families of currents are shown at the top, obtained during steps to potentials ranging from − 20 to − 140 mV in 10 mV intervals, following depolarization pulses at + 40 mV to open (and inactivate) the channels, using the indicated protocol. For clarity, only the first part of the 4-s repolarization steps used to follow the complete decay of the tail currents is shown. The dependence of deactivation rates on repolarization membrane potential is shown at the bottom. Deactivation time constants were quantified by fitting a double exponential to the decaying portion of the tails as described in “Materials and methods” section. Only the magnitude of the deactivation time constant corresponding to the fast decaying major component of current at negative voltages is shown

We also analysed the voltage-dependent deactivation properties of the different splits using a two-step voltage protocol and fitting the tail currents with two exponential functions (see “Materials and methods” section). The data obtained with the different splits are depicted in Fig. 3 and compared with those from wild-type continuous channels. For simplicity, only the time constant of the fast deactivation component that accounts for most of the current amplitude at negative voltages is shown in the lower plot. It is known that Kv11.1 deactivation kinetics become accelerated when different mutations are introduced in the S4–S5 linker [1]. Consistent with previous results, considerably faster rates of voltage-dependent deactivation were observed for the Y545 split as compared with wild-type channels [33]. Surprisingly, in contrast with the accelerated closing rates exhibited by most of the single point mutants introduced in the S4–S5 linker [1], the deactivation kinetics became progressively slowed when the split point was moved from position 545 to 539. Furthermore, the slopes of the time constant versus voltage plots of the splits were smaller than that of the wild-type channel, even producing little voltage-dependent deactivations in those channels broken at the S4 helix/S4–S5 linker connection. Thus, these last channels not only exhibited a strongly left-shifted and very fast non-sigmoidal time course of activation but also a slow and relatively voltage-independent closing phenotype. These data suggest that whereas the energetic barrier between the closed and open state(s) present in the activation pathway of the near-S4 splits is smaller than that of the WT and near-S5 split channels, during the deactivation process in the near-S4 split channels, a destabilization of a transition state leading to an energetic barrier between the open and the closed state(s) bigger that in WT and near-S5 channels is induced.

### Maintenance of functional properties in split channels carrying partial deletions of the S4–S5 linker

**Fig. 4:**
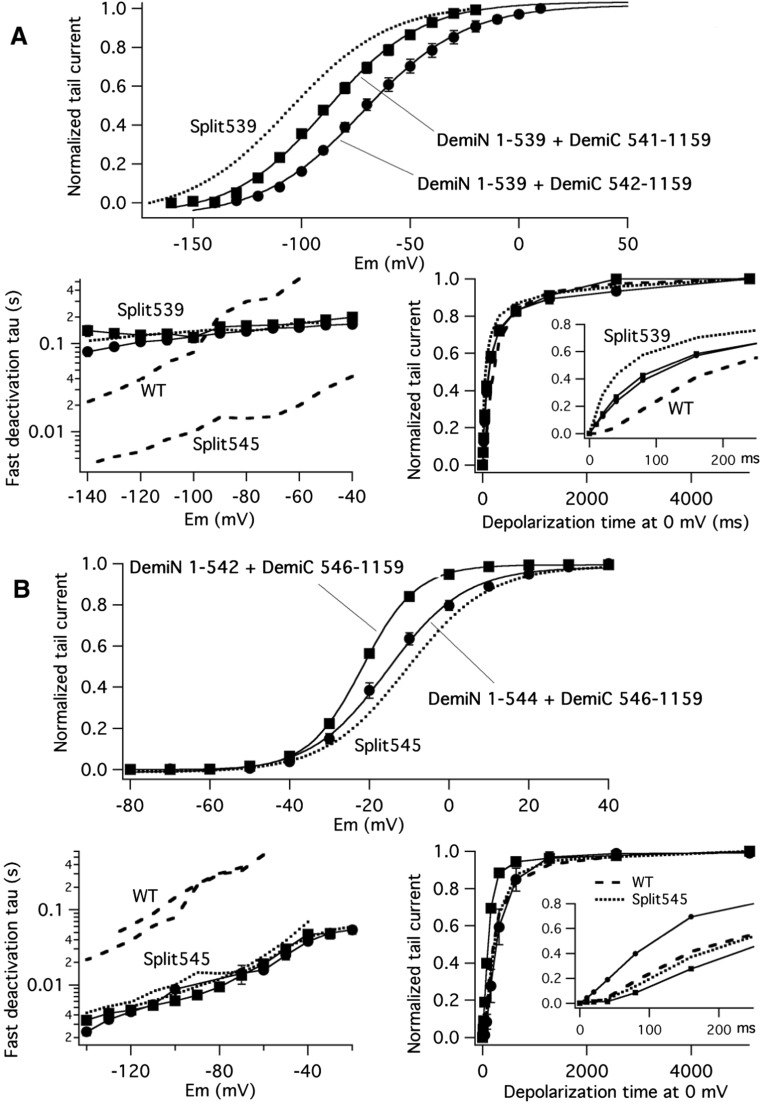
Functional properties of split channels carrying partial S4-S5 linker deletions around the disconnection point between the VSD and the PD. **a** Activation and deactivation gating kinetics of split 539 channels lacking Asp540 or Asp540 plus Arg541 residues. Activation voltage dependence (top). Fractional activation curves were obtained from oocytes co-expressing 1–539 plus 541–1159 or 1–539 plus 542–1159 demi-channel combinations (that will lack D540 or D540 plus R541 residues, respectively), using tail current data as detailed in Fig. 1 for split 539. Continuous lines are Boltzmann curves that best fitted the data as indicated in “Materials and methods” section. A Boltzmann curve from non-deleted split 539 channels as in Fig. 1d is also shown for comparison. Analogy of deactivation kinetics between L539 split channels with and without residues D540 and D540 plus R541 (bottom left). Deactivation time constants were quantified from double exponential fits to the tails as in Fig. 3 (see also “Materials and methods” section). Plots of deactivation time constants for the fast decaying component of the currents as a function of membrane potential are shown. Data from 1 to 539 plus 541–1159 (squares) and 1–539 plus 542–1159 (circles) demi-channel combinations appear superimposed to those from non-deleted split 539 channels (dotted line). Data from continuous wild-type and split 545 channels are also shown as dashed lines for comparison. Comparison of activation rates at 0 mV (bottom right). Averaged plots of normalized tail current magnitudes versus depolarization time at 0 mV are shown from 1 to 539 plus 541–1159 (squares) and 1–539 plus 542–1159 (circles) demi-channel combinations. An expansion of the initial 200-ms time course is shown in the inset. Data from continuous wild-type (dashed line) and non-deleted split 539 channels (dotted line) are also shown for comparison. **b** Activation and deactivation gating kinetics of split 545 channels lacking either Tyr545 or the Ser543 + Glu544 + Tyr545 segment. Analysis of activation voltage dependence (top), deactivation kinetics (bottom left) and the time course of current activation at 0 mV (bottom right) were performed as detailed in **a**. Data from oocytes expressing the 1–544 plus 546–1159 (lacking only the Y545 residue, circles) and the 1–542 plus 546–1159 (lacking the S543 + E544 + Y545 triplet, squares) demi-channel combinations are depicted. Due to the low level of expression obtained with the Y545-deleted construct, a high-K^+^ extracellular solution was used for the 1–542 plus 546–1159 combination recordings (see “Materials and methods” section). Data from continuous wild-type (dotted lines) and non-deleted split 545 channels (dashed lines) are also shown. In both cases, the traces indicating the slowest time constants correspond to data obtained in high-K^+^ extracellular solution

We have previously shown that introduction of an aspartate-to-cysteine mutation at position 540 in the initial part of the S4–S5 linker seems to uncouple the movements of the voltage sensor and the activation gate in Kv11.1 [1], and further evidence of an interaction between the bottom of the S6 helix and residue 540 has been provided by others [17, 50]. Recent data with Kv10.1 channels split at position L341 (equivalent to Kv11.1 split L539), that exhibit a constitutively active phenotype, indicate that the permanently open behaviour of the split is reverted to a normally closing one when the D342 residue (homologous to Kv11.1 D540) immediately after the split point is deleted [49]. Therefore, we also combined different channel halves that, when assembled, give rise to split channels carrying different S4–S5 linker deletions. Unlike previous results with Kv10.1 split channels [33, 49], Kv11.1 splits carrying a deletion covering most of the S4–S5 linker (e.g. co-expressing 1–539 plus 546–1159 or 1–540 plus 546–1159 demi-channels, that would generate split channels lacking 5 or 6 residues of the linker, see Fig. 1a), were not functionally expressed. The results obtained with other functional combinations encompassing split channel complexes with shorter deletions in the N-terminal and the C-terminal regions of the S4–S5 linker are shown in Fig. 4.

Co-expressions of two cRNAs encoding either 1–539 plus 541–1159 or 1–539 plus 542–1159 proteins (that will produce L539 split channels lacking either the D540 residue or the D540 + R541 pair, Fig. 4a) gave rise to functional channels showing little kinetic differences with respect to the full-length L539 split. These split channels maintained a strong negative *V*_1/2_ displacement of the isochronal activation I/V curves albeit slightly shifted to the right (Fig. 4a, *upper panel*), and the same slowed deactivation kinetics when compared to the L539 split (Fig. 4a, *lower left*). Finally, when the activation kinetics were studied at 0 mV using an envelope-of-tails voltage protocol as detailed above, a fast and non-sigmoidal activation time course without an initial delay (such as that of L539 split) was observed in the deleted splits (Fig. 4a, *lower right*). This indicates that the absence of S4–S5 linker’s aspartate 540 and arginine 541 does not greatly modify the functional properties of the split channels that have been disconnected after position 539 at the end of helix S4.

Repeating a similar analysis with channels split at the S4–S5 linker/S5 helix boundary and lacking the last residues of the linker yielded the results shown in Fig. 4b. The kinetic characteristics of an E544 split with a deletion of residue 545 remained almost the same as those of the non-deleted E544 (see above) and Y545 (Fig. 4b) splits. These included a superimposable isochronal activation I/V plot (Fig. 4b, *upper panel*), indistiguishable voltage-dependent deactivation kinetics (Fig. 4b, *lower left*) and a similar activation kinetics at 0 mV (Fig. 4b, *lower right*). Only subtle differences were observed when a Y542 split lacking the S543 + E544 + Y545 triplet (obtained by co-expressing the 1–542 plus 546–1159 demi-channel combination) was studied (Fig. 4b). These included a slightly left-shifted activation I/V plot and faster activation kinetics at 0 mV without initial delay. However, no detectable alterations were observed with respect to the fast deactivation behaviour also exhibited by the Y545 split and the split E544 with a deletion of residue 545. Therefore, apart from a limited effect on activation behaviour, elimination of residues 543 to 545 does not drastically affect the functional characteristics of the splits broken at position 545. Altogether, these data suggest that, despite the short length of the S4–S5 linker in Kv11.1 [60], two different functional outputs are induced when the breaking point is moved along the linker: an alteration of the normal VSD-PD coupling during the activation process mostly induced by breaks in the S4 helix/S4–S5 linker connection and an alteration of the characteristically slow deactivation process, mostly induced by breaks at the C-terminal section of the S4–S5 linker.

### Deactivation slowness in channels split at the beginning of the S4–S5 linker is maintained in a construct carrying a structurally modified N-tail

**Fig. 5:**
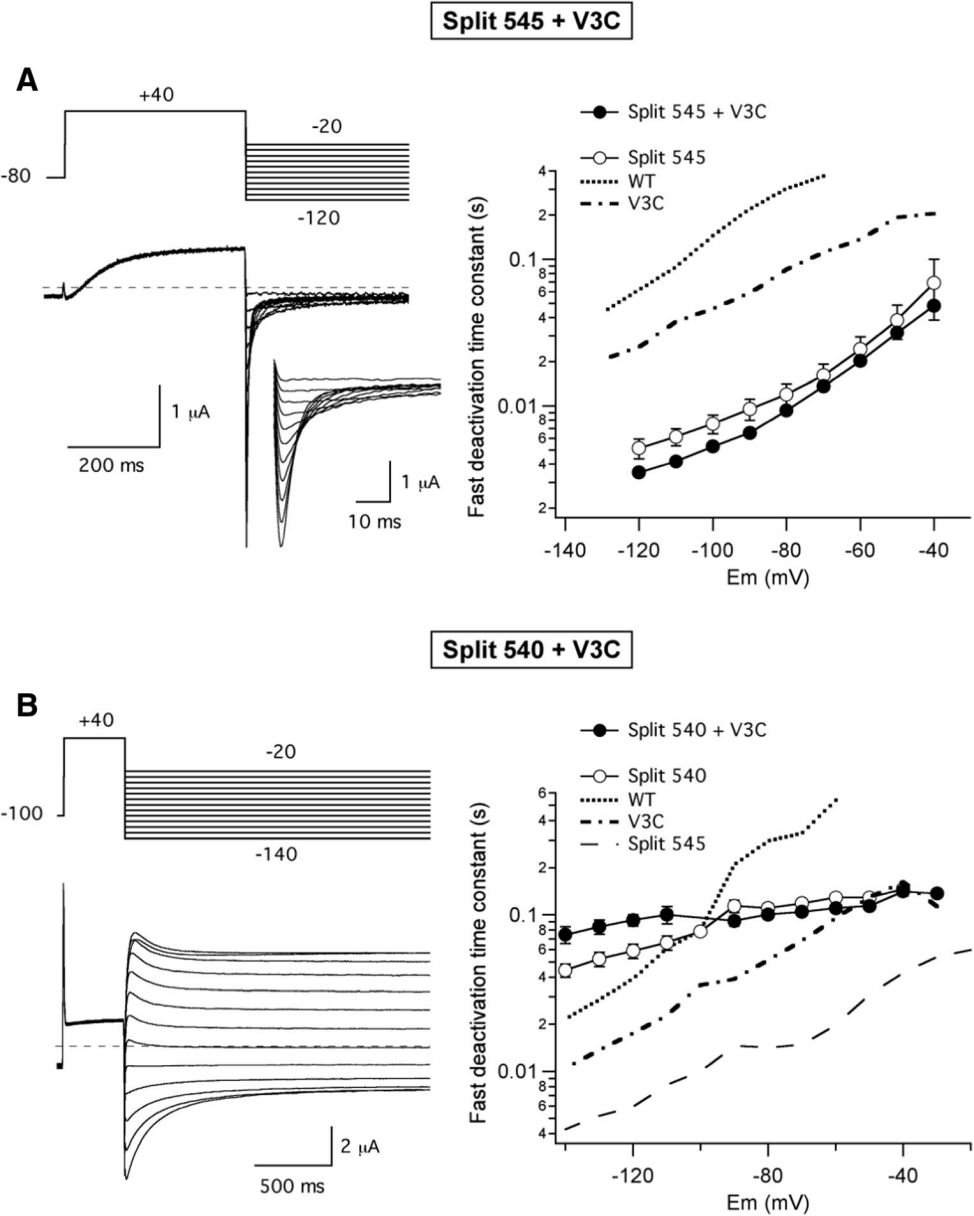
Deactivation kinetics of channels split at the beginning or the end of the S4–S5 linker are not modified by a mutation in residue 3 of the Kv11.1 N-tail. **a** Deactivation characteristics of split 545 channels carrying a V3C mutation in the N-tail of the N-terminal demi-channel. A representative family of currents is shown on the left, obtained with the protocol shown on top of the currents. A high-K^+^ extracellular solution was used to maximize the currents due to the small current magnitudes obtained with the split 545 + V3C construct (see “Materials and methods” section). Only the first part of the 4-s repolarization steps is shown. An expanded view of the initial part of the tails is shown in the inset. The dependence of deactivation rates on repolarization membrane potential is shown on the right. Only the magnitude of the deactivation time constant corresponding to the fast decaying major component of current at negative voltages is shown. Data from WT, the full-length V3C mutant and non-mutated split 545 channels also recorded in high-K^+^ are shown for comparison as indicated. **b** Deactivation characteristics of split 540 channels carrying a V3C mutation in the N-tail of the N-terminal demi-channel. A representative family of currents is shown on the left. The dependence of deactivation rates on repolarization membrane potential is shown on the right. Data from WT, full-length V3C and non-mutated split 540 and split 545 channels are also shown for comparison

It is well known that an important determinant of the characteristic slow deactivation gating of Kv11.1 is the presence of the so-called N-tail at the beginning of the amino terminus and that the influence of this region on deactivation is exerted by its interaction with the channel core probably at the level of the S4–S5 linker [16, 34, 58, 59]. Therefore, we tried to check whether the closing slowness of those channels split near the base of the S4 helix was caused by restoration of such interaction, perhaps lost in those splits cut at the end of the S4–S5 linker. Unfortunately, our repeated attempts to detect currents from Δ2–6, Δ2–16 or Δ2–135 split channels were unsuccessful. Identical negative results were obtained when the size of the N-terminus was increased by insertion of three additional residues. It is important to mention that this lack of functional expression seems to be due to the presence of the deletion/insertion, since we observed currents in the same batches of oocytes injected with cRNAs encoding the splits with an unaltered amino terminus. The molecular and/or cellular reasons for this lack of expression remain to be established. As an alternative analysis, we used functional split channels carrying a V3C single point mutation at the beginning of the N-tail region, known to induce an acceleration of deactivation [11, 16] similar to that induced by deletion either of the whole amino terminus or exclusively of the N-tail, as well as by several single point mutations in the S4–S5 linker [1, 34, 50, 58, 59]. Also, both functional evidences [11, 16] and the direct visualization of a near atomic distance between them in the cryo-EM structure [60] indicate that an interaction exists between the N-tail and the S4–S5 linker of Kv11.1. The results obtained checking the influence of the V3C mutation in the deactivation kinetics of the splits disconnected either at the end or at the beginning of the S4–S5 linker are shown in Fig. 5. It can be observed that the fast voltage-dependent deactivation rate exhibited by the Y545 split channels (see also [33]) remains the same when the demiN-1/545 half of the split carried the V3C mutation (Fig. 5a). It is possible that the very accelerated closing rates induced by the structural alterations in the S4–S5 linker [1, 33] may have concealed any additional decrease in the rates by the mutation. On the other hand, the slow and relatively voltage-independent closing of the D540 split also remained unaltered in the presence of the V3C mutation (Fig. 5b). This indicates that, unlike the situation encountered in the full-length continuous channel, the closing slowness of the channel split near the S4 helix does not rely on a similar interaction between the N-terminal tail and the S4–S5 linker in the channel core. This would explain also why the energetic barrier for activation is maintained low in those constructs cut at the base of the S4 helix (see above), a property also shared by Kv11.1 continuous channels carrying a single point mutation at residue 540 and those lacking the N-terminal tail [1].

### Effect of S4–S5 linker interruptions on Kv11.1 mode shift behaviour

**Fig. 6:**
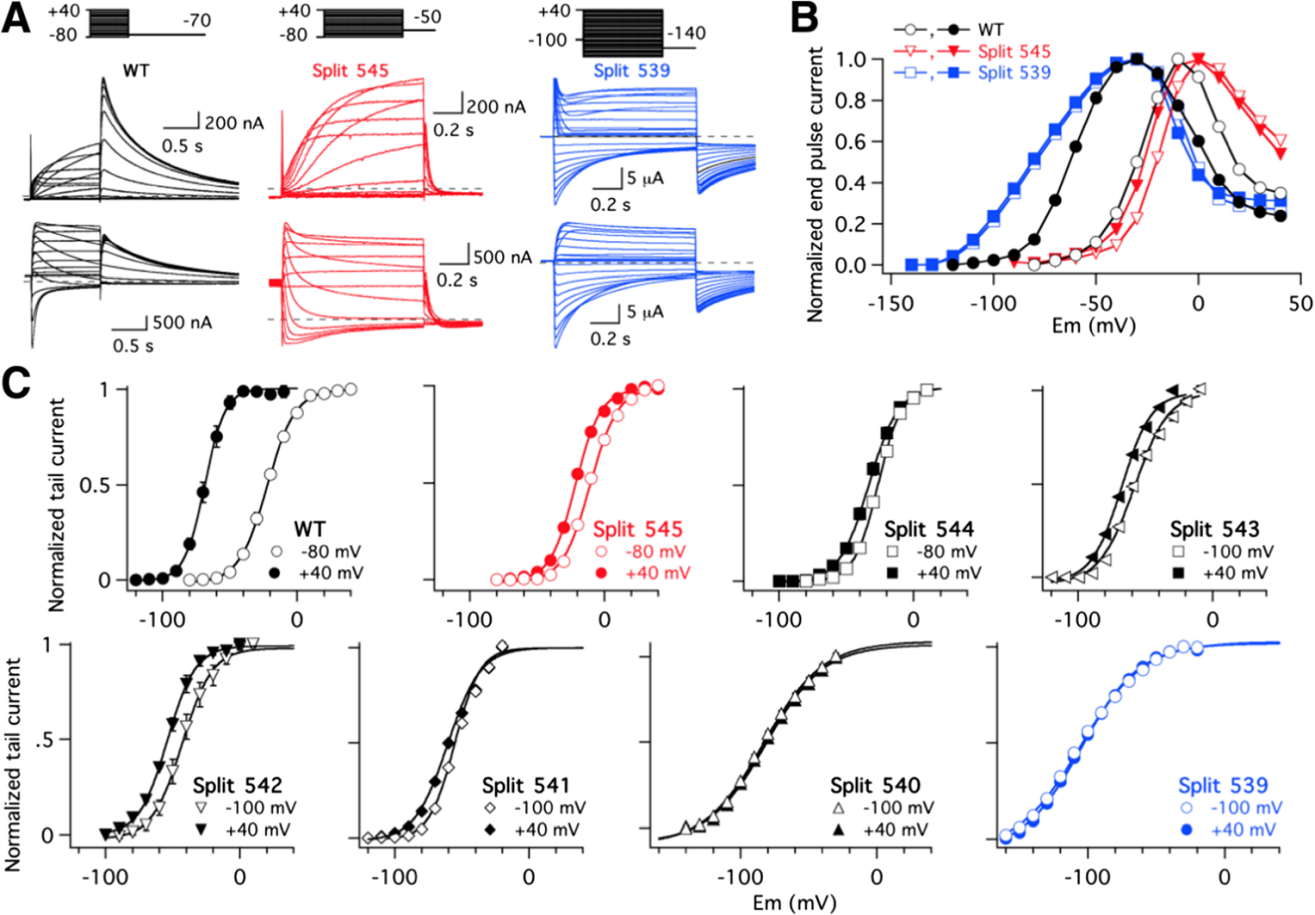
Effect of S4–S5 linker interruptions on Kv11.1 ionic current mode shift. **a** Representative ion currents from continuous wild-type (WT) and splits 545 and 539 studied with the voltage protocols shown at − 80/− 100 (top) and + 40 mV (bottom) holding potentials. **b** Averaged *I* versus *V* relationships from currents measured at the end of the variable depolarization step at different voltages as in panel **a**. Open and closed symbols correspond to data obtained in the same oocytes at hyperpolarized and depolarized holding potentials, respectively. **c** Plots of normalized peak tail current magnitudes as a function of depolarizing voltage for channels split at different positions along the S4–S5 linker. Data from continuous WT channels are also shown for comparison. Averaged values from four to eight cells are shown. Sometimes, error bars are smaller than the symbols. Continuous lines are Boltzmann fits to the data as indicated in “Materials and methods” section. Hyperpolarized (open symbols) and depolarized (closed symbols) holding potential values are indicated in the graphs

As in other voltage-dependent ion channels [22, 66], one remarkable characteristic of Kv11.1 gating is the presence of a prominent mode shift behaviour in which the voltage dependencies of current activation and deactivation are clearly separated [1, 20, 26, 47, 48, 56]. Mode shift has been proposed to be an intrinsic property of the VSD itself, to originate from the mechanical load placed on the VSD by the PD, to involve a rearrangement of the interactions between the cytoplasmic eag and cNBD domains and to be related to C-type inactivation ([10, 20]; see also [66] and references therein). Early data with Kv11.1 indicated that the mode shift is minimized in channels carrying small deletions at the beginning of the N-terminus or mutations in the S4–S5 linker [1], and additional evidence of the involvement of the S4–S5 linker and the amino terminus in the Kv11.1 mode shift has been obtained [20, 26, 47, 48]. Therefore, we also studied the impact of breaking the covalent link between the VSD and the PD at different positions along the S4–S5 linker on mode shift behaviour. The results obtained are summarized in Fig. 6.

Typical examples of current families recorded from Kv11.1 continuous channels and from channels disconnected after residues Y545 and L539 are illustrated in Fig. 6a. Cells were subjected to 1-s depolarization pulses to + 40 mV in 10 mV increments from hyperpolarizing (− 80/− 100 mV) and depolarizing (+ 40 mV) holding potentials, followed by a repolarizing voltage step to negative potentials. Relatively short pulses were chosen to ensure that, apart from the effects due to the intrinsic VSD rearrangements, the regulation of the voltage sensor return and channel closing coming from the pore gate-VSD coupling and load was maintained [20, 48], such that the possible modification of this regulation in the splits could be assessed. A representation of the corresponding I/V plots generated using current magnitude values at the end of the depolarizing test pulses is shown in Fig. 6b. Finally, isochronal G/V relationships at both holding potentials, obtained using tail current measurements of channels split at different points between positions 545 and 539, are shown in Fig. 6c. It can be observed that the − 46 mV shift of G/V *V*_1/2_ value exhibited by the continuous channels is reduced to − 11, − 7, − 10, − 12 and − 5 mV in the splits Y545, E544, S543, Y542 and R541, respectively, and abolished in splits D540 and L539. Therefore, the reduced mode shift exhibited by all the splits disappears when the disconnection is in the N-terminal section of the S4–S5 linker.

### Effect of changing the split point along the S4–S5 linker on voltage-dependent accessibility of a MTS reagent to a cysteine introduced in the upper S4 helix

**Fig. 7:**
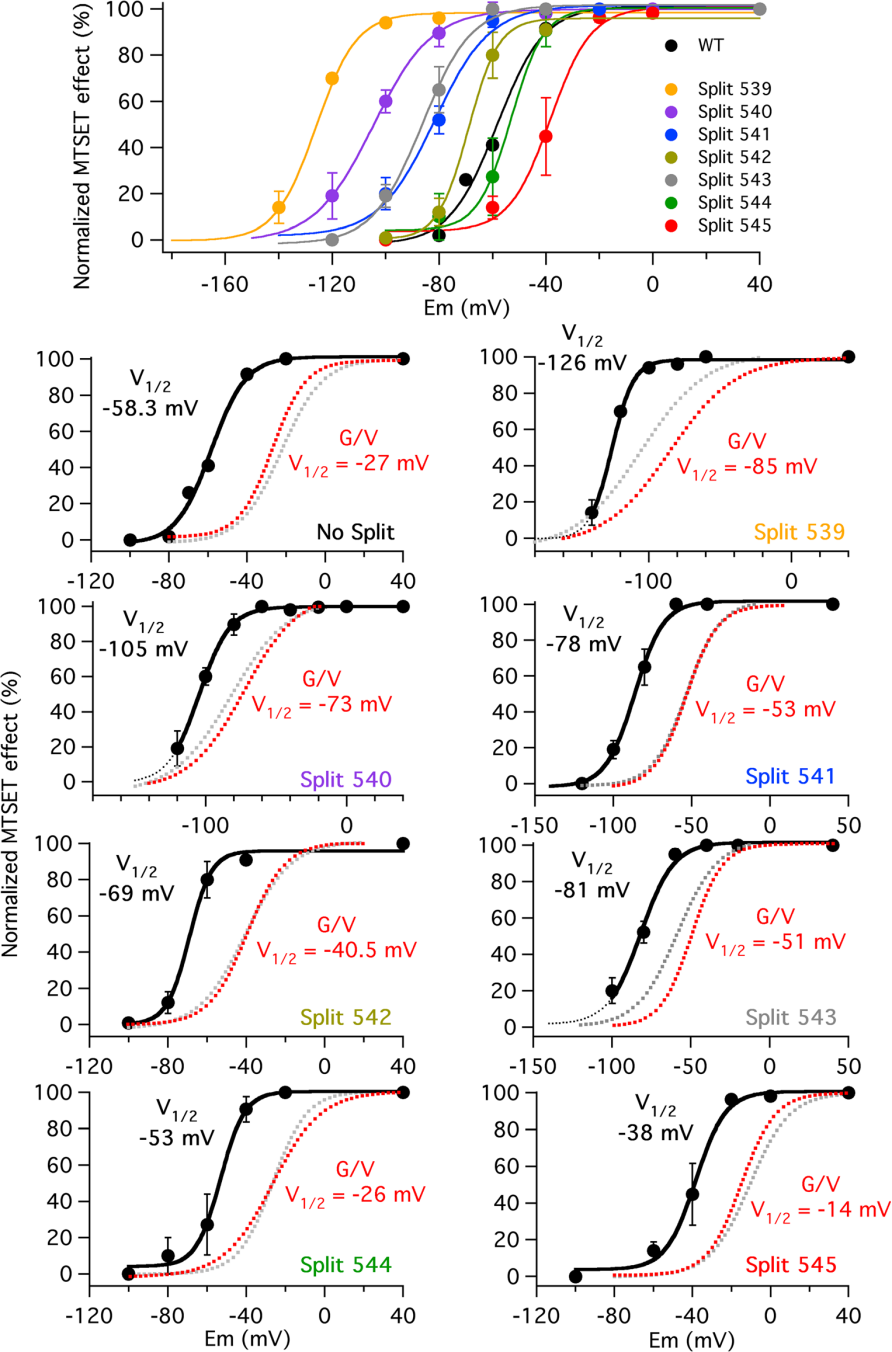
Effect of split position displacement on voltage dependence of MTSET availability to an engineered cysteine at position 521 in the extracellular part of the voltage sensor S4 helix. Summary of voltage dependence of the MTSET effect in different splits carrying the I521C mutation at the extracellular part of the voltage sensor S4 helix, as a function of the holding potential used during the MTS reagent exposure (upper panels). Plots of voltage dependence of the MTSET effect in the individual splits tested (lower panels). Magnitudes of MTSET-induced variations in current kinetics during test ramps as indicated in “Materials and methods” section following a 2-min exposure to 1 mM of the MTS reagent without pulsing at the holding potentials indicated in the abscissa were normalized to those observed at a positive potential value of + 40 mV. Due to the irreversibility of the MTSET effects, only one reagent application was performed and a single holding potential value (followed by a positive control at + 40 mV) was checked in each cell studied. Data from three to six cells were averaged for every single point. Some error bars are smaller than the symbols. Continuous lines correspond to fits using a Boltzmann function as indicated in “Materials and methods” section. The corresponding *V*_1/2_ values are shown on the graphs. *G*/*V* plots from the same constructs obtained from fits to tail current data and *V*_1/2_ values derived from them are also shown in red. Note the similarity of these plots and those from the same splits without the I521C plus C445V and C449V mutations (gray dotted lines; reproduced from Fig. 1d)

One possible explanation for the altered ability of the splits to adequately respond to membrane potential changes is that breaking the covalent connection between the VSD and the PD impairs the voltage sensor conformational changes. Our initial attempts to study by voltage clamp fluorimetry VSD movements using splits labelled with tetramethylrhodamine-5-maleimide (TMRM) in an engineered cysteine at the N-terminal section of the S4 helix [15, 44, 53] were unsuccessful, probably due to insufficient expression levels of the splits. As an alternative, we used methanethiosulfonate (MTSET) to react with and study the exposure and subsequent ion current modification of a cysteine located in the upper S4 helix. For this purpose, a Cys was introduced in position 521 to follow its modification in the presence of MTSET [14, 61], and two endogenous Cys were mutated (C445V and C449V in the S1–S2 linker [14, 15]) to prevent any inadvertent effect caused by their modification by MTSET. As previously reported [14, 15], very little effects on gating were induced by these mutations (Fig. 7; see also below).

As previously described, a 2-min treatment with MTSET of cells expressing continuous I521C mutated channels and held at + 40 mV induced a strong negative shift of the activation voltage dependence (Suppl. Fig. 2A, see [14]). This was manifested as (i) a strongly negatively shifted conductance-to-voltage plot generated with tail current data and (ii) a higher and left-shifted maximum of the I/V plot generated with currents measured at the end of the test pulses used to assess the channel voltage dependence. Indeed, due to the severe impairment of closing caused by trapping cysteine 521 with the MTS reagent, no saturation of the plots obtained in the presence of MTSET was observed, even at the most negative potentials used. The effects of MTSET were not reversed following exhaustive washout of the reagent. It is important to note that unlike the normalized I/V plots previously shown in Fig. 1, total current magnitudes measured at the end of the test pulses are presented here that represent the current window defined by the intersection of the corresponding left-shifted activation and non-shifted inactivation (as in Suppl. Fig. 1) current/voltage plots. This explains the higher and left-shifted maximum obtained, even though a significant alteration of the inactivation kinetics is not taking place.

In order to follow the rates of accessibility of the engineered S4 cysteine to MTSET kinetically and to actively compare the time course of the shifts in voltage dependence, without applying multiple pulses at different potentials to generate the current/voltage plots, we initially used an alternative protocol in which the cells expressing the I521C channels were typically held either at negative (− 100 to − 140 mV for different constructs) or positive (+ 40 mV) potentials, and repetitively pulsed at 5-s intervals using a brief voltage ramp as stimulatory step. As shown in Suppl. Fig. 2B (*right panel*) for a representative experiment performed with the I521C continuous channel, the rates of MTSET-induced modification at + 40 mV followed an exponential time course with a time constant of 49 s. These variations remained the same in cells held at 0 mV, indicating that position 521 becomes exposed to the extracellular membrane-impermeable MTSET at membrane potentials leading to channel activation and inactivation. Since no significant changes in voltage-dependent inactivation properties were induced by the MTSET treatment (see Suppl. Fig. 1C), any contribution of inactivation to the variations observed during the voltage ramp steps can be discarded. Therefore, to overcome the technical problems caused by the high basal K^+^ fluxes present when the membrane is maintained around 0 mV, a holding potential of + 40 mV, at which the channels are mostly inactivated, was routinely used as a reference to study the saturation of the MTSET effects at positive potentials. Repeating the same procedure at a holding potential of − 100 mV, chosen to ensure that the I521C continuous channel remained closed between pulses until the MTSET locked them open, yielded the results shown in the *left panel* of Suppl. Fig. 2B. In this case, a delayed and much slower effect of the reagent was obtained, with an approximate time constant of 169 s. Interestingly, changing the holding potential to + 40 mV after several minutes of treatment accelerated the development of the MTSET effects, that followed an exponential time course with a time constant (41 s) analogous to that previously obtained without the initial period at − 100 mV.

Almost the same results were obtained when a channel split at the S4–S5 linker/S5 helix interface was studied (split 545, Suppl. Fig. 3Aa). Strikingly, repeating the analysis with channels split at position 539 of the S4 helix/S4–S5 linker boundary indicated that the MTSET effect followed an almost identical time course when they were held open/inactivated at + 40 mV and when a very negative holding potential of − 140 mV (necessary to hold mainly closed this left-shifted channel construct) was used (Suppl. Fig. 3Ba). In this case, due to the very limited effects of the reagent in position, magnitude and voltage-dependent shift of the peak currents during the ramps, only the variations in rectification factor of the ramp-induced currents were used to evaluate this construct.

At first, these results seemed to suggest a very little or no accessibility to the cysteine engineered at position 521 of the S4 helix in the continuous and the Y545 splits and, in contrast, a higher availability of the sulfhydryl moiety of the introduced residue in the L539 split. However, since the time expended in the activated state(s) during the repeated pulses could be different in the different constructs, particularly for those strongly shifted to the left, we wanted to be sure that the divergent results were not biased by the pulsing protocol used. This prompted us to check the magnitude of the MTSET-induced effects on channels held at a negative potential without the influence of the repetitive pulses. The Suppl. Fig. 2C shows the results obtained with the continuous channel and Suppl. Figs. 3Ab and 3Bb those obtained with the splits Y545 and L539 after a 2-min MTSET treatment without pulsing. In this case, a holding potential of − 100 mV was used with the continuous and split 545 channels, whereas the L539 split was maintained at − 140 mV (the most negative potential at which, although only in a reduced sample of cells, a stable recording was achieved). As an internal control, a second period at a holding potential of + 40 mV was used to quantify in every individual cell the maximum MTSET effect. Providing that enough negative potential is used to maintain the channels mostly deactivated, no significant effects of MTSET took place, indicating that without repetitive pulsing, the I521C residue is in all cases inaccessible to this reagent at rest. Indeed, almost identical results were obtained when the same procedure was applied to non-pulsed D540 split channels held at − 120 mV and to R541, Y542, S543 and E544 splits held at − 100 mV. Therefore, we checked next the voltage dependence of the MTSET effect without repetitive pulsing in the different splits. As can be observed in the upper panel of Fig. 7, similar to the gradual alterations in current activation voltage dependence summarized in Fig. 1, when the split point was displaced from position 545 to 539, a gradual shift to more hyperpolarized potential values was induced on the voltage dependence of the MTSET effect. Interestingly, in all cases, the *V*_1/2_ values of this effect were significantly left-displaced, as compared to those corresponding to the conductance-to-voltage plots of the same constructs (lower panels in Fig. 7), suggesting that the voltage sensor S4 movement is being tracked in the MTSET experiments [15, 19, 20, 41, 61]. Furthermore, unlike the behaviour of the G/V curves previously described (see Fig. 1 above), the voltage-dependent availability to MTSET showed similar *z*_*g*_ values for all tested splits, including those (e.g. splits 539 and 540) interrupted at the end of the S4 helix. Thus, these values amounted 3.0, 3.0, 2.5, 3.8, 2.9, 4.1, 2.4 and 3.2 for WT and splits 545 to 539, respectively. Altogether, these data indicate that the voltage dependence shifts induced by changing the split position along the S4–S5 linker are also accompanied by a shift in the voltage-dependent availability of the I521C residue to the MTS reagent. Our data demonstrate for the first time that changing the covalent connection between the voltage sensor and the pore domains impacts the structural reorganizations and motion of the VSD. Moreover, since the S4–S5 linker acts as an integrator of signals coming from other cytoplasmic channel domains [1, 35, 67], they suggest that the final consequences of the S4–S5 linker breaks on gating also depend of the bidirectional influence of these intracellular domains on the VSD, and vice versa.

## Discussion

In this report, we present findings concerning how voltage-dependent gating takes place in Kv11.1 channels lacking a covalent link between VSD and PD (split channels). Thus, we show for the first time that when the break point is progressively displaced from the carboxy to the amino end of the S4–S5 linker (see Fig. 1A; [60, 62]) to the base of transmembrane segment S4, a shift to the left in the voltage dependence and a more negative Δ*G*_0_ for current activation is correspondingly induced. Also, channels split at the base of S4 helix present an increased destabilization of closing, a reduced ability to reach more distal closed state(s) and a reduced voltage dependency of both activation and deactivation gating. On the other hand, our data suggest that the VSD to PD coupling during activation is quite normal in the splits interrupted at the C-terminal section of the S4–S5 linker that, consistently with previous results with channels carrying distinct structural alterations in the linker [1], only seemed to show a markedly accelerated deactivation behaviour. However, it appears that a loose coupling between the VSD and the channel gate is present when the disconnection is in the N-terminal section of the linker near the S4 helix. We have also found that the typical Kv11.1 mode shift, manifested as a clear separation of the voltage dependencies of current activation and deactivation, is either reduced or completely abolished in channels split at the end or the beginning of the S4–S5 linker, respectively. Our results also indicate that a combination of demi-channels that would generate a Kv11.1 split without the complete S4–S5 linker was not functionally expressed. However, deletion of the residues located at the beginning of the linker does not significantly modify the functional characteristics of the channels interrupted at the end of S4 helix. Also, in splits broken at the end of the linker, deletion of the C-terminal residues essentially reproduces the behaviour of the non-deleted splits. Surprisingly, we found that even small structural modifications of the channel N-terminal tail obliterated the functional expression of the splits, suggesting that the N-tail is important in generating functional split channels. Finally, based on data obtained with the substituted cysteine accessibility method [25, 29], we found that when the split point was displaced from the end to the beginning of the S4–S5 linker, a gradual shift to more hyperpolarized potentials is induced on the voltage dependence of MTSET accessibility to an engineered Cys residue located in the upper S4 helix of the VSD. Thus, the negative shifts in channel activation voltage dependence caused by moving the split point along the S4–S5 linker were accompanied by equivalent shifts in the voltage dependence of S4 motion across the membrane. Interestingly, our results showing clear differences in the voltage-dependent behaviour of the voltage sensor in the different splits, contrast with those of a recent study with Kv10.1, in which no difference on the voltage dependency of voltage sensor motion was observed, even in those channels disconnected near the S4 helix that show a constitutively active phenotype [49]. Methodological differences apart, perhaps in the constitutively active split Kv10.1 channels, an almost completely pore-uncoupled voltage sensor leads to a sensor exhibiting conformational rearrangements similar to those of the WT channel, whereas in the left-shifted but still partially coupled Kv11.1 splits, the gating maintains a more strict dependence on S4 motion. Further work will be necessary to understand the molecular reason(s) of these differences. In any event, our results show that disconnections between the VDS and PD cause a significant impact in the structural reorganizations of the voltage sensor.

It has been indicated that, unlike the situation encountered with “atypical” prokaryotic ion channels in which expressing the isolated PD leads to a more open pore (see [8] and references therein), in eukaryotic channels, an intrinsically closed state can be favoured in the virtual absence of VSDs [28, 64]. However, in channels such as Kv11.1, an intrinsically more stable open state has been proposed [1, 17, 23, 55], this being compatible with (i) a situation in which VSDs exert work to close the pore [7, 8, 31, 55], (ii) a “more constitutive” conductance induced in response to a loose VSD/PD coupling [7, 31, 52, 55] and (iii) the fact that the channel essentially “falls” into the open state when the transmembrane electrical field (e.g. at 0 mV) disappears [36]. Our data demonstrating that an intact S4 helix/S4–S5 linker connection is essential for a stable closing state add further support to this idea. This would be also coherent with the requirement demonstrated here of a considerable amount of energy (− 120/− 140 mV), to efficiently close those splits (e.g. 539 and 540 splits) in which the VSD/PD connection seems to be particularly altered, leading to a relatively uncoupled split channel that remains predominantly opened. Although in these cases the voltage dependence of gating still follows that of the sensor rearrangements, it is tempting to speculate that the behaviour of these splits is governed by their need to use a “quite inefficient” VSD to close them, whereas small reductions of the voltage gradient rapidly release them to an intrinsically stable open state [5, 36, 55]. A relatively inefficient and/or partially uncoupled VSD will be also consistent with the reduced voltage dependence of gating exhibited by these constructs and their significantly smaller amount of equivalent gating charges (*z*_*g*_ values) estimated from the slope of the G/V curves, but not from the voltage-dependent availability to the MTS reagent that tracks the movement of the S4 sensor helix. It is also possible that, as previously indicated [31], the job performed by the VSD is dual to both close and open the channel if both protein states represent an energetic minimum. Alternatively, since expression of an isolated PD from the same split channel does not yield any detectable current, it is possible that (providing that the Kv11.1 PD alone normally assembles and traffics to the membrane as demonstrated with that of Kv10.1 [49]), the energetic profile of the isolated PD favouring a more stable closed conformation is different from that of the complete VSD/PD assembly, that favours a preferentially opened basal conformation [66].

In addition to their effects on activation and deactivation voltage dependencies, lack of a covalent link between the VSD and the PD either reduces the isochronal ion current mode shift magnitude in the splits interrupted at the end of the S4–S5 linker or completely abolishes it when the cut is near the S4 helix. In the first case, the disconnection between VSD and PD did not greatly influence activation gating but strongly accelerated deactivation, and in the second case, severely impaired activation and significantly slowed deactivation. This suggests that the split-related gating mode shift reduction is not exclusively caused by an accelerated deactivation [48], since the strongest reductions were observed with 539/540 splits exhibiting a markedly slowed closing. An important role of the N-terminal regions and of rearrangement of the cytoplasmic eag-cNBD domain interactions in KCNH channels mode shift behaviour has been recognized [10, 20]. Also, the participation of the S4–S5 linker in the Kv11.1 mode shift has been demonstrated [26, 47, 48, 53]. Therefore, it is probable that alterations in the S4–S5 linker, or in any of the components pertaining to the dual pathway recently proposed for Kv10.1 to connect the gating assembly located below the VSD/S4–S5 linker/gate interface with the gating components in the channel core [67], could allosterically affect different phases of an otherwise non-electromechanical gating mechanism [33]. This could include the short-term and long-term reorganizations of the VSD leading to the mode shift [10, 20, 47, 48, 66]. In this scenario, the existence of a dynamic network of interactions involving the N-terminal tail, the S4–S5 linker and the C-terminal portion of the S6 and also other cytoplasmic regions such as the PAS domain or the C-linker/cNBD regions could be particularly relevant [6, 7, 11–13, 16], constituting an essential component of the Kv11.1 gating machinery itself or an important regulator of the gating process. Thus, we propose that some of this ensemble of interactions is differentially affected when the split position is moved along the S4–S5 linker. Nevertheless, additional measurements of these putative interactions would be required to clarify the molecular details of the modifications triggered by the channel splits on the gating mechanism(s).

Although the structural changes in the splits affecting the sensitive VSD-PD interface may modify the coupling properties, the S4–S5 split channels also provide some clues for understanding voltage-dependent gating in Kv11.1 and other members of the KCNH family. Indeed, the data reported here add supporting functional evidence for some of the predictions based on the recent cryo-EM structure of Kv10.1 and Kv11.1, regarding an alternative gating mechanism for the KCNH channels [60, 62]. In this context, two main questions may be considered next: (i) what molecular mechanism could more adequately explain the gating characteristics observed in the different splits and (ii) how this mechanism can be reconciled with our present knowledge of the molecular architecture of the KCNH channels.

**Fig. 8:**
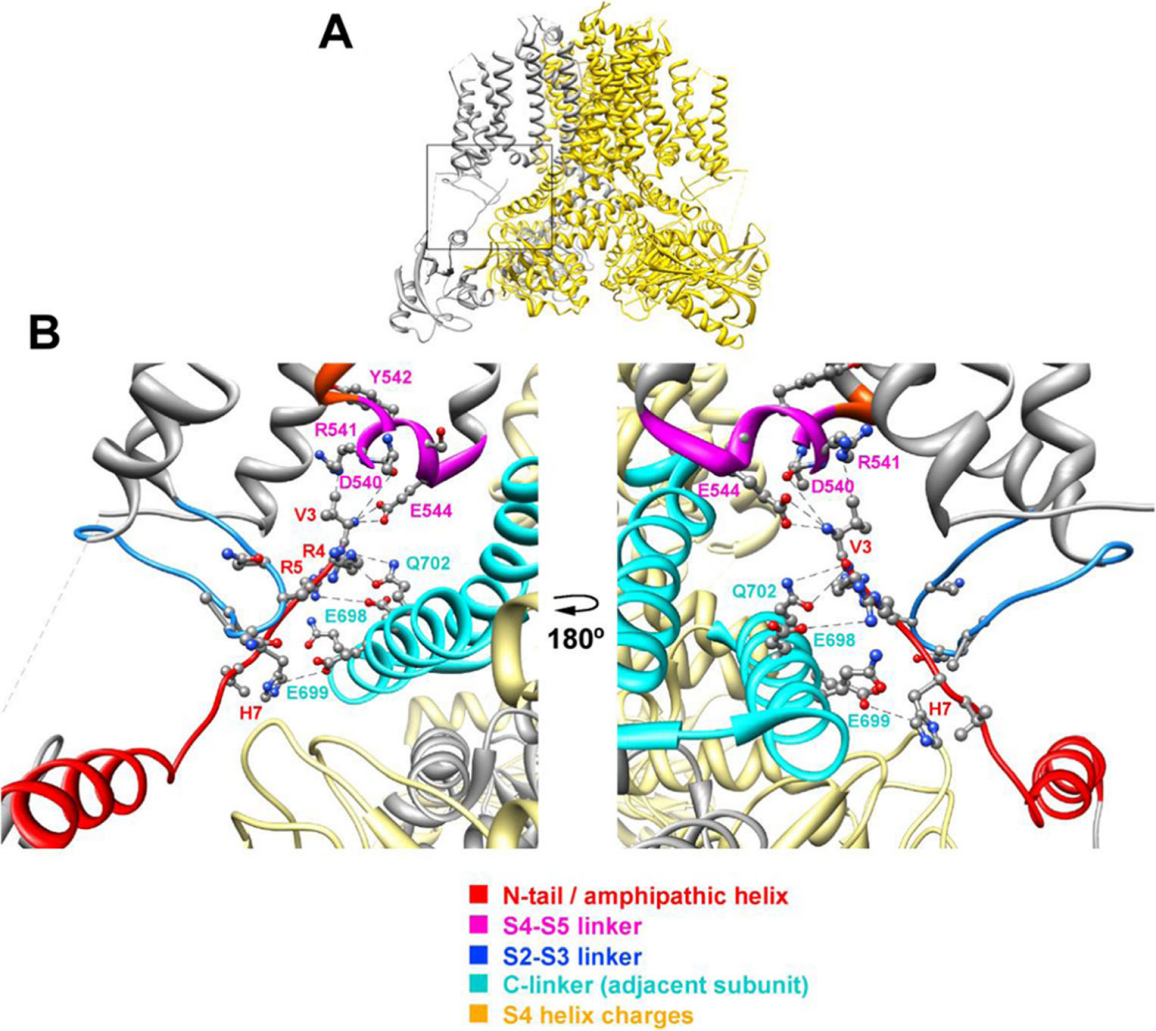
Close apposition and interactions of the Kv11.1 N-tail with the S4–S5, S2–S3 and C-linkers of the channel. **a** Lateral view of the Kv11.1 tetrameric structure ([60] PDB code 5VA2). A highlighted subunit is coloured in gray. **b** Enhanced view of the region inside the signalled square in **a** with ribbons corresponding to the indicated domains and residue symbols coloured as shown at the bottom. Lateral chains of the selected residues are shown as ball and stick with oxygen and nitrogen atoms as red and blue spheres. Dashed lines indicate interatomic distances ranging between 3.0 and 5.4 Å. Structures were processed with UCSF Chimera [39]

As well as the previous functional data obtained with split channels [33], the cryo-EM structure of Kv10.1 and Kv11.1 indicates that, unlike Kv 1 to 9 channels, the KCNH family members possess a very short S4–S5 linker that is not domain swapped [60, 62] and cannot work as a mechanical lever to gate the channel. We have previously suggested that the intrinsic flexibility of the N-terminal tail and its location close to both the S4–S5 and the C-linkers constitute a crucial factor for modulation of Kv11.1 gating [6, 11–13, 16]. Strikingly, a positioning of the N-tail in close contact with the S4–S5 and the C-linkers has been subsequently confirmed by the cryo-EM structure of Kv11.1 in which a distance no longer than 5 Å between several residues corresponding to these regions has been demonstrated in a direct way (Fig. 8). Our functional data allow us to propose that in the splits, there are some interactions in which the channel N-tail acts as a coupling factor between the base of the voltage sensor and the PD participating, not only in the modulation of gating but also in the voltage-dependent electro-allosteric mechanism that couples the VSD reorganizations to the operation of the PD gate. Some evidence in support of this hypothesis is reported here. Thus, whereas the C-terminal half of the S4–S5 linker seems to be crucial for setting the deactivation properties of the channels, the initial section near the S4 helix plays a fundamental role in VSD-PD coupling during the activation process. This would be consistent not only with the “relatively uncoupled” phenotype exhibited by those Kv11.1 splits disconnected at residues 539 and 540 but also by the demonstrated generation of a constitutively active channel when the Kv10.1 sequence is interrupted in the equivalent position [49]. Interestingly, in addition to demonstrating that the Kv11.1 N-tail establishes direct contacts with the S4–S5 and the C-linkers, the cryo-EM structure points to the long S2–S3 linker (a conserved feature of the KCNH family [62]) as an additional component of the aforementioned interactional gating network (Fig. 8 [60]). Experiments are currently in process to check the possible involvement of this loop in the Kv11.1 gating process(es).

In summary, we propose an integrative model for Kv11.1 gating in which by actively pushing the C-terminal end of the S4 helix and/or the initial section of the S4–S5 linker against the S5–S6 module [33, 62], the closing of an otherwise intrinsically stable open pore can be favoured at negative transmembrane potentials. Relatively slow structural reorganizations of the VSD upon depolarization would release its constraints on the PD leading to channel opening, a process that is further slowed down [20] by the necessary removal of interactions between the N-terminus and the gating machinery. This scenario is what comes mostly affected by the break in the S4 helix/S4–S5 linker connection as demonstrated here and, indirectly, when the flexible S4–S5/S5 hinge is made more rigid ([53], see also [9]). Arguably, disruptions towards the N-terminus of the linker will also influence S4 mobility and the general structural reorganizations of the VSD as shown here, since the role of the S4–S5 linker as an anchor of sorts and its interactions with putative intracellular tethers would be impaired. Increasing the strength of the transmembrane electric field at negative voltages leads to channel closing, a process also delayed by a second set of interactions between the N-terminus and the channel core. This process, that is particularly influenced by breaks at the end of the S4–S5 linker, is induced by dynamic reallocation of the N-terminal tail position during the activation step(s) [47]. Therefore, besides its contribution to better understanding the molecular mechanisms that explain the gating properties of the splits, our work may provide some additional clues for a better understanding of the role of regions functionally involved in VSD-PD coupling in Kv11.1 and other voltage-dependent potassium channels which, when altered, lead to a number of disorders, such as long QT syndrome, episodic ataxia and epilepsy [5, 42].

## Electronic supplementary material

ESM 1 (DOCX 875 kb)

## References

[1] Alonso-Ron C, de la Peña P, Miranda P, Domínguez P, Barros F (2008). Thermodynamic and kinetic properties of amino-terminal and S4-S5 loop HERG channel mutants under steady-state conditions. Biophys J.

[2] Barros F, Domínguez P, de la Peña P (2012). Cytoplasmic domains and voltage-dependent potassium channel gating. Front Pharmacol.

[3] Barros F, Gómez-Varela D, Viloria CG, Palomero T, Giráldez T, de la Peña P (1998). Modulation of human erg K^+^ channel gating by activation of a G protein-coupled receptor and protein kinase C. J Physiol.

[4] Bezanilla F (2008). How membrane proteins sense voltage. Nat Rev Mol Cell Biol.

[5] Blunck R, Batulan Z (2012). Mechanism of electromechanical coupling in voltage-gated potassium channels. Front Pharmacol.

[6] Cheng YM, Claydon TW (2012). Voltage-dependent gating of hERG potassium channels. Front Pharmacol.

[7] Choveau FS, Abderemane-Ali F, Coyan FC, Es-Salah-Lamoureux Z, Baro I, Loussouarn G (2012). Opposite effects of the S4-S5 linker and PIP(2) on voltage-gated channel function: KCNQ1/KCNE1 and other channels. Front Pharmacol.

[8] Chowdhury S, Chanda B (2012). Thermodynamics of electromechanical coupling in voltage-gated ion channels. J Gen Physiol.

[9] Chowdhury S, Haehnel BM, Chanda B (2014). Interfacial gating triad is crucial for electromechanical transduction in voltage-activated potassium channels. J Gen Physiol.

[10] Dai G, Zagotta WN (2017). Molecular mechanism of voltage-dependent potentiation of KCNH potassium channels. elife.

[11] De la Peña P, Alonso-Ron C, Machín A, Fernández-Trillo J, Carretero L, Domínguez P, Barros F (2011). Demonstration of physical proximity between the amino terminus and the S4–S5 linker of the hERG potassium channel. J Biol Chem.

[12] De la Peña P, Machín A, Fernández-Trillo J, Domínguez P, Barros F (2013). Mapping of interactions between the amino and carboxy termini and the channel core in hERG K^+^ channels. Biochem J.

[13] De la Peña P, Machín A, Fernández-Trillo J, Domínguez P, Barros F (2015). Interactions between the N-terminal tail and the gating machinery of hERG K^+^ channels both in closed and open/inactive states. Pflugers Arch.

[14] Dou Y, Goodchild J, Vander Velde R, Wu Y, Fedida D (2013). The neutral, hydrophobic isoleucine at position I521 in the extracellular S4 domain of hERG contribuyes to channel gating equilibrium. Am J Phys.

[15] Es-Salah-Lamoureux Z, Fougere R, Xiong PY, Robertson GA, Fedida D (2010). Fluorescence-tracking of activation gating in human ERG channels reveals rapid S4 movement and slow pore opening. PLoS One.

[16] Fernández-Trillo J, Barros F, Machín A, Carretero L, Domínguez P, de la Peña P (2011). Molecular determinants of interactions between the N-terminal domain and the transmembrane core that modulate hERG K^+^ channel gating. PLoS One.

[17] Ferrer T, Rupp J, Piper DR, Tristani-Firuozi M (2006). The S4-S5 linker directly couples voltage sensor movement to the activation gate in the human ether-a-go-go-related gene (hERG) K^+^ channel. J Biol Chem.

[18] Gómez-Varela D, de la Peña P, García J, Giráldez T, Barros F (2002). Influence of amino-terminal structures on kinetic transitions between several closed and open states in human erg K^+^ channels. J Membrane Biol.

[19] Goodchild SJ, Fedida D (2014). Gating charge movement precedes ionic current activation in hERG channels. Channels.

[20] Goodchild SJ, Macdonald LC, Fedida D (2015). Sequence of gating charge movement and pore gating in hERG activation and deactivation pathways. Biophys J.

[21] Gutman GA, Chandy KG, Grissmer S, Lazdunski M, McKinnon D, Pardo LA, Robertson GA, Rudy B, Sanguinetti MC, Stühmer W, Wang X (2005). International Union of Pharmacology. LIII. Nomenclature and molecular relationships of voltage-gated potassium channels. Pharmacol Rev.

[22] Haddad GA, Blunck R (2011). Mode shift of the voltage sensors in Shaker K^+^ channel is caused by energetic coupling to the pore domain. J Gen Physiol.

[23] Hardman RM, Stansfeld PJ, Dalibalta S, Sutcliffe MJ, Mitcheson JS (2007). Activation gating of hERG potassium channels: S6 glycines are not required as gating hinges. J Biol Chem.

[24] Hille B (2001). Ion channels of excitable membranes.

[25] Horn R (1998). Explorations of voltage-dependent conformational changes using cysteine scanning. Meth Enzymol.

[26] Hull CM, Sokolov S, Van Slyke AC, Claydon TW (2014). Regional flexibility in the S4-S5 linker regulates hERG channel closed-state stabilization. Pflugers Arch.

[27] Isacoff EY, Jan LY, Minor DL (2013). Conduits of life’s spark: a perspective on ion channel research since the birth of neuron. Neuron.

[28] Jensen MO, Jogini V, Borhani DW, Leffler AE, Dror RO, Shaw DE (2012). Mechanism of voltage gating in potassium channels. Science.

[29] Karlin A, Akabas MH (1998). Substituted-cysteine accessibility method. Meth Enzymol.

[30] Kiehn J, Lacerda AE, Brown AM (1999). Pathways of HERG inactivation. Am J Phys.

[31] Labro AJ, Snyders DJ (2012). Being flexible: the voltage-controllable activation gate of Kv channels. Front Pharmacol.

[32] Liu S, Rasmusson RL, Campbell DL, Wang S, Strauss HC (1996). Activation and inactivation kinetics of an E-4031-sensitive current from single ferret atrial myocytes. Biophys J.

[33] Lorinczi E, Gomez-Posada JC, de la Peña P, Tomczak AP, Fernandez-Trillo J, Leipscher U, Stuhmer W, Barros F, Pardo LA (2015). Voltage-dependent gating of KCNH potassium channels lacking a covalent link between voltage-sensing and pore domains. Nat Commun.

[34] Ng CA, Hunter MJ, Perry MD, Mobli M, Ke Y, Kuchel PW, King GF, Stock D, Vandenberg JI (2011). The N-terminal tail of hERG contains an amphipatic α-helix that regulates channel deactivation. PLoS One.

[35] Ng CA, Perry MD, Tan PS, Hill AP, Vandenberg JI (2012). The S4–S5 linker acts as a signal integrator for hERG K^+^ channel activation and deactivation gating. PLoS One.

[36] Patlak JB (1999). Cooperating to unlock the voltage-dependent K channel. J Gen Physiol.

[37] Perry MD, Ng C, Mann SA, Sadrieh A, Imtiaz M, Hill AP, Vandenberg JI (2015). Getting to the heart of hERG K^+^ channel gating. J Physiol.

[38] Perry MD, Wong S, Ng CA, Vandenberg JI (2013). Hydrophobic interactions between the voltage sensor and pore mediate inactivation in Kv11.1 channels. J Gen Physiol.

[39] Pettersen EF, Goddard TD, Huang CC, Couch GS, Greenblatt DM, Meng EC, Ferrin TE (2004). UCSF Chimera-avisualization system for exploratory research and analysis. J Comput Chem.

[40] Piper DR, Hinz WA, Talluri CK, Sanguinetti MC, Tristani-Firuozi M (2005). Regional specificity of human ether-a-go-go-related gene channel activation and inactivation gating. J Biol Chem.

[41] Piper DR, Varghese A, Sanguinetti MC, Tristani-Firouzi M (2003). Gating currents associated with intramembrane charge displacement in HERG potassium channels. Proc Natl Acad Sci USA.

[42] Sanguinetti MC (2010). HERG1 channelopathies. Pflugers Arch.

[43] Smith PL, Baukrowitz T, Yellen G (1996). The inward rectification mechanism of the HERG cardiac potassium channel. Nature.

[44] Smith PL, Yellen G (2002). Fast and slow voltage sensor movements in HERG potassium channels. J Gen Physiol.

[45] Subbiah RN, Clarke CE, Smith DJ, Zhao J, Campbell TJ, Vandenberg JI (2004). Molecular basis of slow activation of the human ether-a-go-go related gene potassium channel. J Physiol.

[46] Swartz KJ (2008). Sensing voltage across lipid membranes. Nature.

[47] Tan PS, Perry MD, Ng CA, Vandenberg JI, Hill AP (2012). Voltage-sensing domain mode shift is coupled to the activation gate by the N-terminal tail of hERG channels. J Gen Physiol.

[48] Thouta S, Hull CM, Shi YP, Sergeev V, Young J, Cheng YM, Claydon TW (2017). Stabilization of the activated hERG channel voltage sensor by depolarization involves the S4-S5 linker. Biophys J.

[49] Tomczak AP, Fernández-Trillo J, Bharill S, Papp F, Panyi G, Stühmer W, Isacoff EY, Pardo LA (2017). A new mechanism of voltage-dependent gating exposed by Kv10.1 channels interrumpted between voltage sensor and pore. J Gen Physiol.

[50] Tristani-Firuozi M, Chen J, Sanguinetti MC (2002). Interactions between S4-S5 linker and S6 transmembrane domain modulate gating of HERG K^+^ channels. J Biol Chem.

[51] Trudeau MC, Warmke JW, Ganetzky B, Robertson GA (1995). HERG, a human inward rectifier in the voltage-gated potassium channel family. Science.

[52] Upadhyay SK, Nagarajan P, Methew MK (2009). Potassium channel opening: a subtle two-step. J Physiol.

[53] Van Slyke AC, Rezazadeh S, Snopkowski M, Shi P, Allard CR, Claydon TW (2010). Mutations within the S4–S5 linker alter voltage sensor constraints in hERG K^+^ channels. Biophys J.

[54] Vandenberg JI, Perry MD, Perrin MJ, Mann SA, Ke Y, Hill AP (2012). hERG K^+^ channels: structure, function, and clinical significance. Physiol Rev.

[55] Vardanyan V, Pongs O (2012). Coupling of voltage sensors to the channel pore: a comparative view. Front Pharmacol.

[56] Viloria CG, Barros F, Giráldez T, Gómez-Varela D, de la Peña P (2000). Differential effects of amino-terminal distal and proximal domains in the regulation of human erg K^+^ channel gating. Biophys J.

[57] Wang S, Liu S, Morales MJ, Strauss HC, Rasmusson RL (1997). A quantitative analysis of the activation and inactivation kinetics of HERG expressed in Xenopus oocytes. J Physiol.

[58] Wang J, Trudeau MC, Zappia AM, Robertson GA (1998). Regulation of deactivation by an amino terminal domain in human ether-á-go-go-related gene potassium channels. J Gen Physiol.

[59] Wang J, Myers CD, Robertson GA (2000). Dynamic control of deactivation gating by a soluble amin-terminal domain in HERG K^+^ channels. J Gen Physiol.

[60] Wang W, MacKinnon R (2017). Cryo-EM structure of the open human ether-a-go-go-related K^+^ channel hERG. Cell.

[61] Wang Z, Dou Y, Goodchild SJ, Es-Salah-Lamoureux Z, Fedida D (2013). Components of gating charge movement and S4 voltage-sensor exposure during activation of hERG channels. J Gen Physiol.

[62] Whicher JR, MacKinnon R (2016). Structure of the voltage-gated K^+^ channel eag1 reveals an alternative voltage sensing mechanism. Science.

[63] Yellen G (2002). The voltage-gated potassium channels and their relatives. Nature.

[64] Yifrach O, MacKinnon R (2002). Energetics of pore opening in a voltage-gated K^+^ channel. Cell.

[65] Yu FH, Catterall WA (2004). The VGL-chanome: a protein superfamily specialized for electrical signalling and ionic homeostasis. Sci STKE.

[66] Zhao J, Blunck R (2016). The isolated voltage sensing domain of the Shaker potassium channel forms a voltage-gated cation channel. elife.

[67] Zhao Y, Goldschen-Ohm MP, Morais-Cabral JH, Chanda B, Robertson GA (2017). The intrinsically liganded cyclic nucleotide-binding homology domain promotes KCNH channel activation. J Gen Physiol.

