# Supplementary material for: Gating mechanism of Kv11.1 (hERG) K+ channels without covalent connection between voltage sensor and pore domains
Source: Pflugers Arch. 2017 Dec 21;470(3):517–36. doi: 10.1007/s00424-017-2093-9 (PMC5805800; doi:10.1007/s00424-017-2093-9)

**Supplementary Figures**

**Suppl. Fig 1.** Minimal changes in the voltage dependence of inactivation are caused by either S4-S5 excisions or I521C channels treatment with MTSET. (A) Comparison of voltage-dependence of inactivation from continuous wild-type (WT) and Y545 split channels. Families of current traces recorded in response to the triple pulse protocol illustrated at the top, are shown on the left. Due to the strongly accelerated closing of the Y545 split channels, conditioning voltage steps of very short duration (10 ms) were used to determine the isochronal inactivation level preventing virtual disappearance of the current at relatively small negative membrane potentials. Uncorrected (open symbols) and corrected for deactivation (closed symbols) inactivation voltage-dependence plots are shown on the right (see also Methods). Note the relative concordance between the uncorrected and corrected data in the case of the WT channels due to the small closing level reached during the short conditioning steps. (B) Comparison of steady-state voltage-dependence of inactivation from continuous WT and L539 split channels. 20 ms conditioning steps at different voltages were used. Uncorrected (open symbols) and corrected for deactivation (closed symbols) inactivation voltage-dependence plots are shown on the right. Note the concordance between the uncorrected and corrected data in the case of the slowly deactivating L539 split channels. (C) Comparison of steady-state voltage-dependence of inactivation from continuous I521C channels before (open symbols) and after (closed symbols) treatment with MTSET. Families of current traces recorded in the absence (Control) and the presence of 1 mM MTSET are shown on the left. Inactivation voltage-dependence plots are shown on the right. Due to minimization of closing in the presence of the MTS reagent, only control current data are shown corrected for deactivation (closed symbols).

**Suppl. Fig 2.** MTSET modification of I521C continuous Kv11.1 channels. (A) Effect of MTSET on activation voltage dependence. *Left.* Representative current traces were obtained with the voltage protocol shown at the top before (Control) and after 2 min of treatment with 1 mM MTSET. Cells were held at +40 mV during MTSET perfusion. *Right*. Current-voltage (I/V) relationships obtained at the end of the voltage pulses (circles in the current traces on the left) before and after MTSET are shown at the top. Plots of normalized peak tail current magnitudes (squares on the left) are shown at the bottom. Curve superimposed to the control data represents a Boltzmann fit. No fit was performed for MTSET data because the conductance did not saturate up to the most negative voltage value tested. In this case, the minimal current level was arbitrarily set to zero. (B) Estimation of MTSET-induced modification rates of cysteine 521. Current traces shown in the insets were obtained in response to 1,700 ms voltage ramps between -120 and +50 mV, preceded by a conditioning 500 ms step to -120 mV and followed by a 1 s step to the same voltage. This protocol was repeated at 5 s intervals. Current traces corresponding to times immediately before application of 1 mM MTSET (black), at the end of the MTSET exposure using a holding potential (H.P.) of -100 mV (green), and after the MTSET treatment at a holding potential of +40 mV (red), are depicted. Plots illustrate the time course of MTSET-induced modifications normalized to those observed at the end of the treatment at +40 mV. The changes in the position of the peak current during the voltage ramp were quantified for the plots (see Methods). Similar results were obtained following the increase in the peak current magnitude during the ramp, the variation of the amount of tail current recorded at the end of the -120 voltage step included immediately before or after the ramps, and the changes in the rectification factor during the ramps (see Methods). Mono-exponential fits to the data are shown superimposed to the symbols. The values of the corresponding time constants (tau) are indicated in the graphs. Note the delayed and slow development of the MTSET effect at -100 mV and that for this reason the indicated tau only represents an approximate value. (C) Absence of MTSET effects in cells held at negative potentials no submitted to repetitive depolarization pulsing. Current traces obtained in response to the indicated voltage protocols are shown. No pulses were applied during the 2 min periods indicated by black boxes at which the cells were continuously held at the indicated potential.

**Suppl. Fig 3.** MTSET modification of split channels carrying the I521C mutation. (A) Split 545. (Aa) Estimation of MTSET-induced modification rates of cysteine 521. Current traces shown in the insets and time course plots of MTSET-induced modifications were obtained as detailed in the legend of Suppl. Fig. 2. Note the very limited effects in the tail current magnitudes at the end of the repolarizing step after the ramp probably due to the still marked and accelerated closing behaviour exhibited by this construct after the treatment with MTSET. For this reason, only the variations in the magnitude and positioning of the peak currents or in the rectification factor during the ramps (see Methods) were quantified to assess the MTSET effects on this construct. (Ab) Absence of MTSET effects in cells held at negative potentials no submitted to repetitive depolarization pulsing. Current traces obtained in response to the indicated voltage protocols are shown. No pulses were applied during the 2 min periods indicated by black boxes at which the cells were continuously held at the indicated potential. (B) Split 539. (Ba) MTSET-induced modification rates of cysteine 521. Current traces shown in the insets were obtained in response to 1,700 ms voltage ramps between -140 and +50 mV, preceded by a conditioning 500 ms step at -140 mV and followed by a 1 s period tat the same voltage. This protocol was repeated at 5 s intervals. Current traces corresponding to times immediately before application of 1 mM MTSET (black), at the end of the MTSET exposure using a holding potential (H.P.) of -140 mV (green), and after the MTSET treatment at a holding potential of +40 mV (red), are depicted. Plots illustrate the time course of MTSET-induced modifications normalized to those observed at the end of the treatment at +40 mV. Due to the very limited effects of MTSET in the position and magnitude of the peak currents during the ramps, only the decrease in the ratio of the slopes (rectification factor) during the steeper raising phase and the initial minimum slope phase of the ramp-induced currents were quantified for the plots. (Bb) Absence of MTSET effects in cells held at negative potentials no submitted to repetitive depolarization pulsing. Current traces obtained in response to the indicated voltage protocol are shown. No pulses were applied during the 2 min periods indicated by black boxes at which the cells were continuously held at the indicated potential. Note the maintenance of the outward rectification during the initial phase of the ramp at a holding potential of -140 mV and its absence once the cell was held at +40 mV.


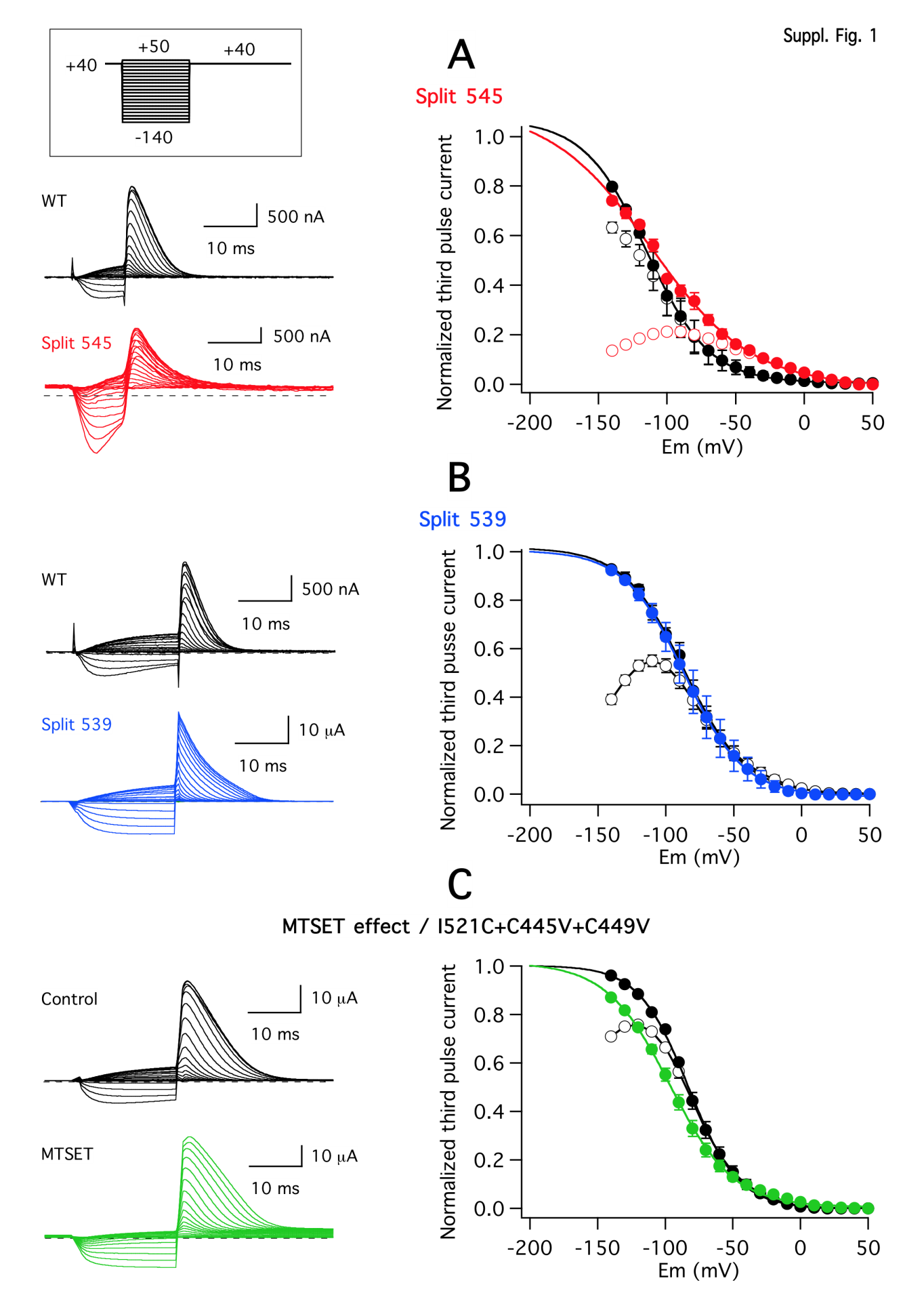


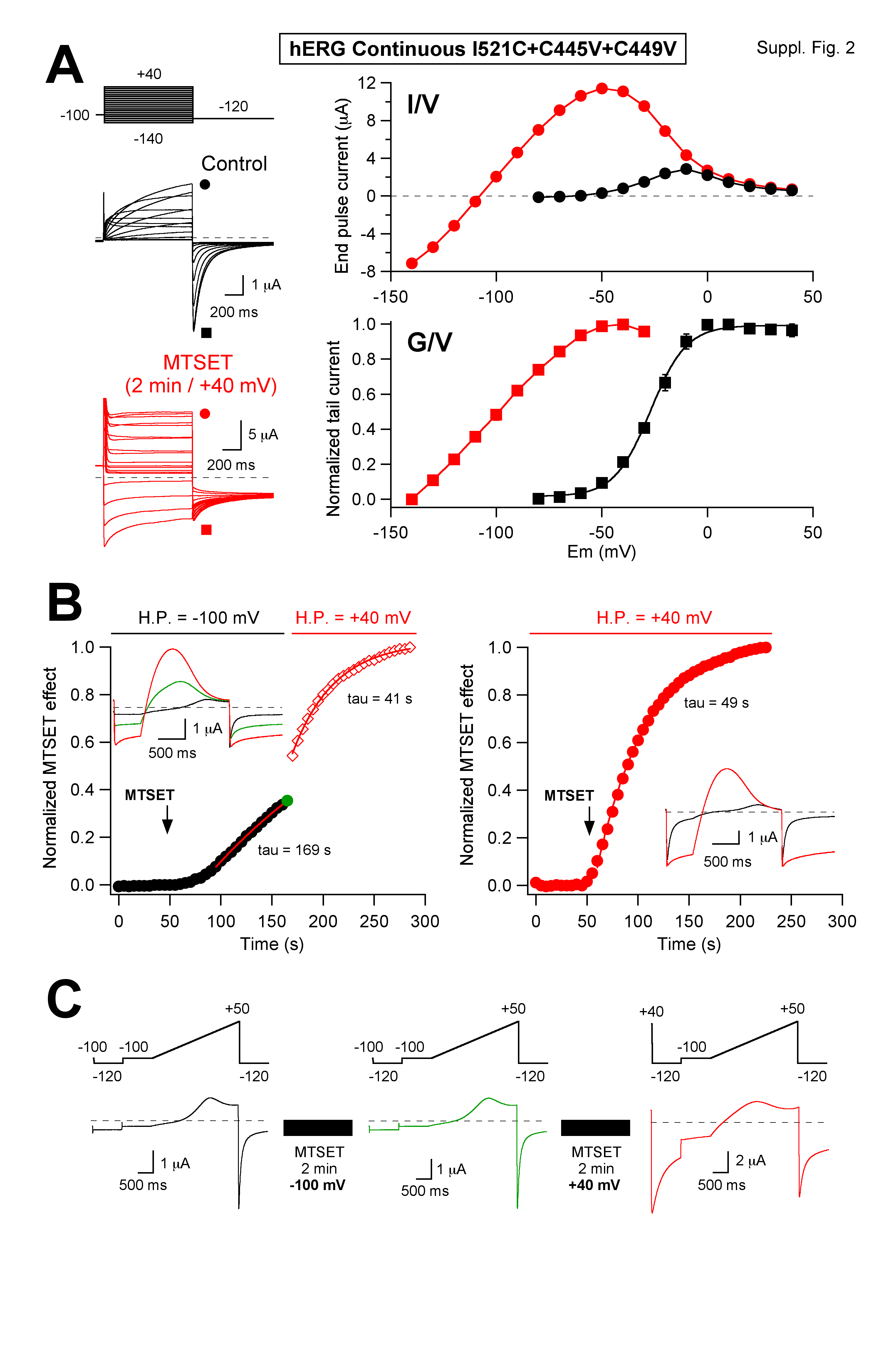


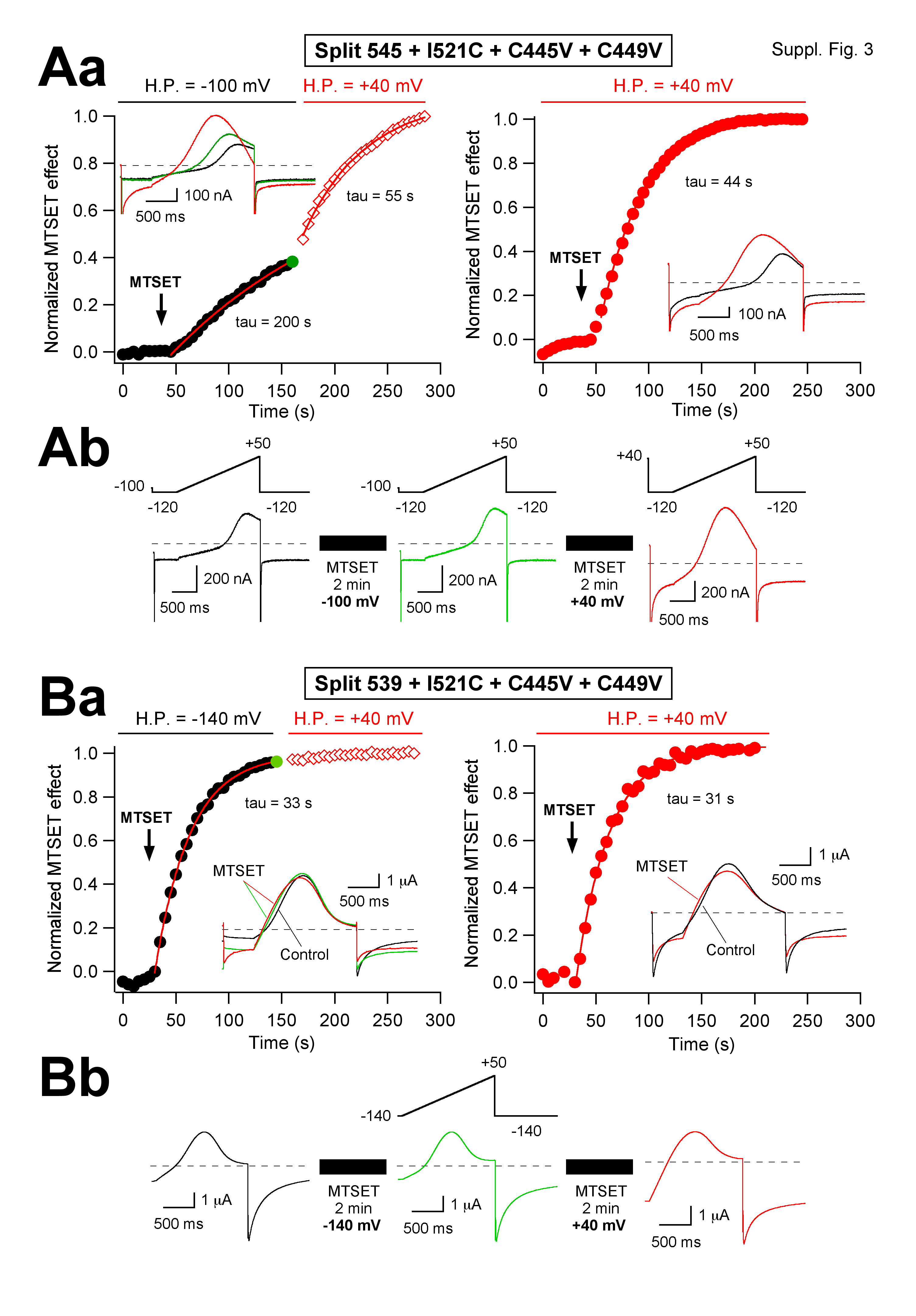

Supplement: Supplementary file 1 — (DOCX 875 kb) [file 424_2017_2093_MOESM1_ESM.docx]
